# Circulating metabolomic markers linking diabetic kidney disease and incident cardiovascular disease in type 2 diabetes: analyses from the Hong Kong Diabetes Biobank

**DOI:** 10.1007/s00125-024-06108-5

**Published:** 2024-02-27

**Authors:** Qiao Jin, Eric S. H. Lau, Andrea O. Luk, Claudia H. T. Tam, Risa Ozaki, Cadmon K. P. Lim, Hongjiang Wu, Elaine Y. K. Chow, Alice P. S. Kong, Heung Man Lee, Baoqi Fan, Alex C. W. Ng, Guozhi Jiang, Ka Fai Lee, Shing Chung Siu, Grace Hui, Chiu Chi Tsang, Kam Piu Lau, Jenny Y. Leung, Man-wo Tsang, Elaine Y. N. Cheung, Grace Kam, Ip Tim Lau, June K. Li, Vincent T. F. Yeung, Emmy Lau, Stanley Lo, Samuel Fung, Yuk Lun Cheng, Chun Chung Chow, Weichuan Yu, Stephen K. W. Tsui, Brian Tomlinson, Yu Huang, Hui-yao Lan, Cheuk Chun Szeto, Wing Yee So, Alicia J. Jenkins, Erik Fung, Mirthe Muilwijk, Marieke T. Blom, Leen M. ‘t Hart, Juliana C. N. Chan, Ronald C. W. Ma, on behalf of the Hong Kong Diabetes Biobank Study Group

**Affiliations:** 1 Department of Medicine and Therapeutics, The Chinese University of Hong Kong, Prince of Wales Hospital, Hong Kong, China; 2 Hong Kong Institute of Diabetes and Obesity, The Chinese University of Hong Kong, Hong Kong, China; 3 Li Ka Shing Institute of Health Sciences, The Chinese University of Hong Kong, Hong Kong, China; 4 CUHK-SJTU Joint Research Centre in Diabetes Genomics and Precision Medicine, Hong Kong Institute of Diabetes and Obesity, The Chinese University of Hong Kong, Hong Kong, China; 5 School of Public Health (Shenzhen), Sun Yat-sen University, Shenzhen, Guangdong China; 6 Department of Medicine and Geriatrics, Kwong Wah Hospital, Hong Kong, China; 7 Diabetes Centre, Tung Wah Eastern Hospital, Hong Kong, China; 8 Diabetes and Education Centre, Alice Ho Miu Ling Nethersole Hospital, Hong Kong, China; 9 North District Hospital, Hong Kong, China; 10 Department of Medicine and Geriatrics, Ruttonjee Hospital, Hong Kong, China; 11 Department of Medicine and Geriatrics, United Christian Hospital, Hong Kong, China; 12 Tseung Kwan O Hospital, Hong Kong, China; 13 Department of Medicine, Yan Chai Hospital, Hong Kong, China; 14 Centre for Diabetes Education and Management, Our Lady of Maryknoll Hospital, Hong Kong, China; 15 Department of Medicine, Pamela Youde Nethersole Eastern Hospital, Hong Kong, China; 16 Department of Medicine and Geriatrics, Princess Margaret Hospital, Hong Kong, China; 17 Department of Medicine, Alice Ho Miu Ling Nethersole Hospital, Hong Kong, China; 18 Department of Electronic and Computer Engineering, The Hong Kong University of Science and Technology, Hong Kong, China; 19 School of Biomedical Sciences, The Chinese University of Hong Kong, Hong Kong, China; 20 Faculty of Medicine, Macau University of Science and Technology, Macau, China; 21 Department of Biomedical Sciences, City University of Hong Kong, Hong Kong, China; 22 NHMRC Clinical Trials Centre, University of Sydney, Sydney, NSW Australia; 23 Baker Heart and Diabetes Institute, Melbourne, VIC Australia; 24 Department of Epidemiology and Data Science, Amsterdam UMC - Location Vrije Universiteit Amsterdam, Amsterdam, the Netherlands; 25 Health Behaviors & Chronic Diseases Research Program, Amsterdam Public Health, Amsterdam UMC, Amsterdam, the Netherlands; 26 Department of General Practice, Amsterdam UMC - Location Vrije Universiteit Amsterdam, Amsterdam, the Netherlands; 27 Department of Biomedical Data Sciences, Section Molecular Epidemiology, Leiden University Medical Centre, Leiden, the Netherlands; 28 Department of Cell and Chemical Biology, Leiden University Medical Centre, Leiden, the Netherlands

**Keywords:** Cardiovascular disease, Diabetic kidney disease, Metabolomics, NMR spectroscopy, Prognostic biomarker, Risk stratification, Severely increased albuminuria, Type 2 diabetes

## Abstract

**Aims/hypothesis:**

The aim of this study was to describe the metabolome in diabetic kidney disease (DKD) and its association with incident CVD in type 2 diabetes, and identify prognostic biomarkers.

**Methods:**

From a prospective cohort of individuals with type 2 diabetes, baseline sera (*N*=1991) were quantified for 170 metabolites using NMR spectroscopy with median 5.2 years of follow-up. Associations of chronic kidney disease (CKD, eGFR<60 ml/min per 1.73 m^2^) or severely increased albuminuria with each metabolite were examined using linear regression, adjusted for confounders and multiplicity. Associations between DKD (CKD or severely increased albuminuria)-related metabolites and incident CVD were examined using Cox regressions. Metabolomic biomarkers were identified and assessed for CVD prediction and replicated in two independent cohorts.

**Results:**

At false discovery rate (FDR)<0.05, 156 metabolites were associated with DKD (151 for CKD and 128 for severely increased albuminuria), including apolipoprotein B-containing lipoproteins, HDL, fatty acids, phenylalanine, tyrosine, albumin and glycoprotein acetyls. Over 5.2 years of follow-up, 75 metabolites were associated with incident CVD at FDR<0.05. A model comprising age, sex and three metabolites (albumin, triglycerides in large HDL and phospholipids in small LDL) performed comparably to conventional risk factors (C statistic 0.765 vs 0.762, *p*=0.893) and adding the three metabolites further improved CVD prediction (C statistic from 0.762 to 0.797, *p*=0.014) and improved discrimination and reclassification. The 3-metabolite score was validated in independent Chinese and Dutch cohorts.

**Conclusions/interpretation:**

Altered metabolomic signatures in DKD are associated with incident CVD and improve CVD risk stratification.

**Graphical Abstract:**

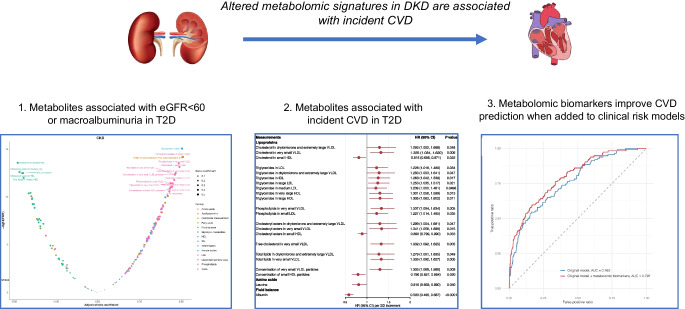

**Supplementary Information:**

The online version of this article (10.1007/s00125-024-06108-5) contains peer-reviewed but unedited supplementary material.


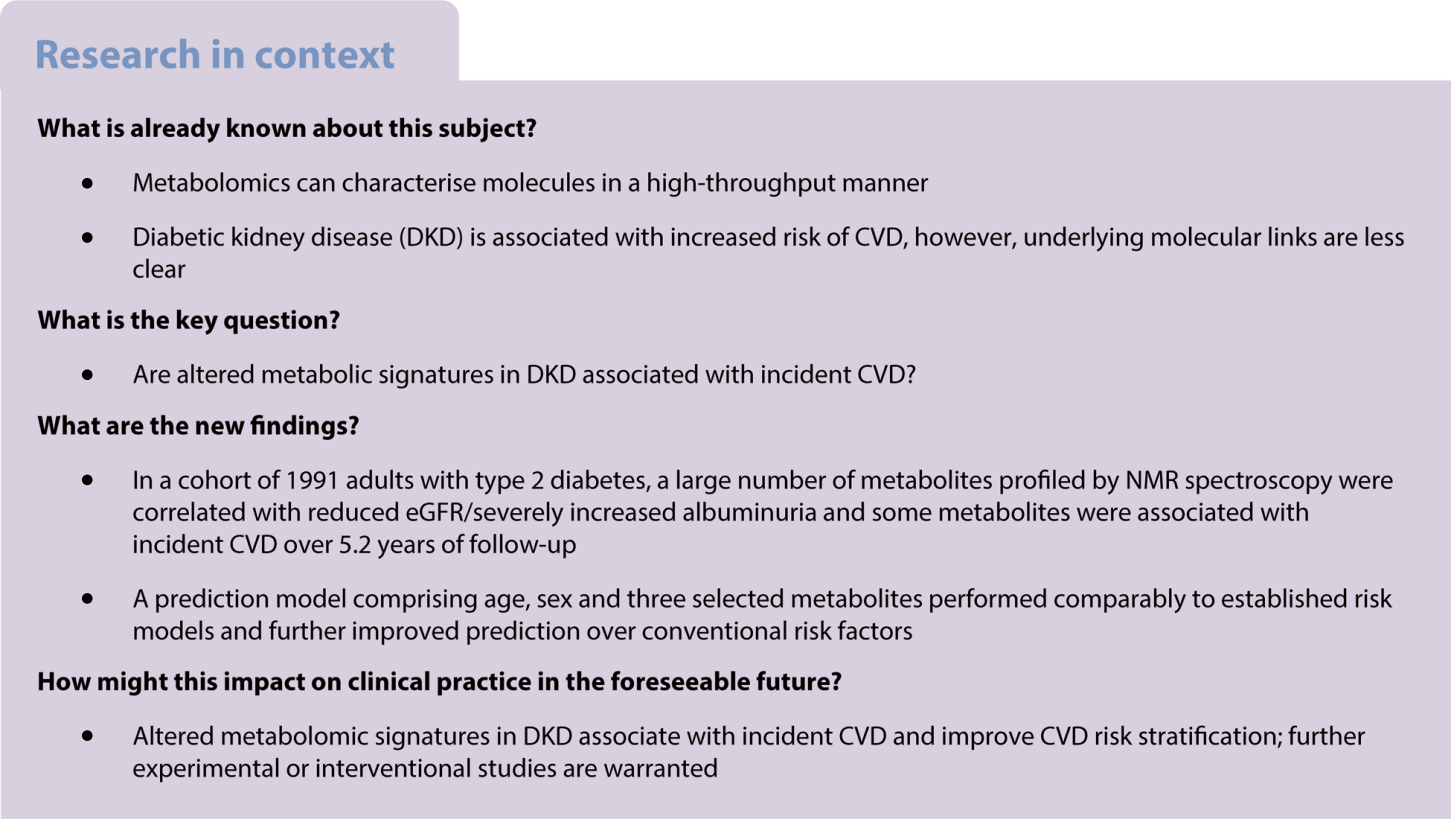


## Introduction

Chronic kidney disease (CKD) develops in approximately 40% of people with type 2 diabetes [1] and is associated with increased risk of CVD and mortality [2]. Diabetes is associated with two- to fourfold increased risk of CVD [3], whereas higher CVD incidence was reported in people with CKD than in diabetes [4], suggesting particularly high CVD risk in diabetic kidney disease (DKD). Despite multifactorial management and agents with pleiotropic cardiorenal benefits, DKD prognosis remains poor.

Type 2 diabetes is characterised by atherogenic dyslipidaemia: elevated triglyceride-rich lipoproteins (TRLs) and reduced HDL, contributing to substantial residual risk despite optimal LDL-cholesterol (LDL-C) levels [5]. In CKD, TRLs are increased owing to impaired lipoprotein lipase activities and diminished clearance caused by altered apolipoprotein C-3 (ApoC-3) metabolism [6]. The major structural protein of TRLs, apolipoprotein B (ApoB), can flux across endothelium and be trapped in the artery wall, initiating atherosclerosis by releasing cholesterol to macrophages [7]. Beyond lipids, the kidney also can regulate circulating metabolites via filtration, reabsorption, secretion, catabolism and anabolism [8]. With advances in technologies, metabolites can be quantified simultaneously in a high-throughput manner and multiple metabolites have been associated with DKD [8–10].

Higher TRLs, ApoB, phenylalanine, inflammation markers and lower HDL and apolipoprotein A-1 (ApoA-1) have been associated with decreased eGFR in people with type 2 diabetes [9], and replicated in a larger study [10], indicating that altered lipoprotein and metabolic profiles may reflect impaired kidney function in diabetes. Furthermore, TRLs, ApoB and phenylalanine have been associated with CVD in people with CKD or type 2 diabetes [11–13], suggesting that the altered metabolome in DKD may partly explain the increased CVD risk. Although the causal relation between the metabolites and CVD in people with DKD is not yet fully understood, Mendelian randomisation studies have suggested TRLs and ApoB are causally associated with CVD [14, 15]; phenylalanine has been associated with type 2 diabetes [16], impaired kidney function [9], heart failure [17] and CVD [18] in large cohort studies. Better understanding of the potential metabolic links between DKD and CVD is therefore warranted.

Herein, we investigated the metabolomic signature of DKD and examined its association with incident CVD in a well-characterised prospective cohort of individuals with type 2 diabetes. Metabolomic biomarkers were selected among metabolites associated with CVD and were evaluated for their prognostic value towards CVD prediction. External validation of the identified biomarkers for incident CVD was performed in Chinese [19] and Dutch cohorts [20].

## Methods

### Study population

#### The Hong Kong Diabetes Biobank

The Hong Kong Diabetes Biobank (HKDB) is a multicentre prospective cohort study, coordinated by the Chinese University of Hong Kong. The study design, recruitment methods, collection of baseline data and biochemical investigations have been published [21–24]. Briefly, HKDB used similar enrolment and assessment methods to that of the Hong Kong Diabetes Register (HKDR, based at the Prince of Wales Hospital [PWH], the teaching hospital of the Chinese University of Hong Kong), incorporating comprehensive and structured assessment of risk factors and diabetes complications [23]. At enrolment, participants consented for prospective follow-up (until death) and biobanking of blood samples for research. All participants provided written informed consent and the study was approved by the Joint Chinese University of Hong Kong-New Territories East Cluster Clinical Research Ethics Committee and the Clinical Research Ethics Committee of each participating hospital.

#### HKDR and the Hoorn Diabetes Care System cohort

HKDR has been briefly described above [19]. The Hoorn Diabetes Care System (DCS) cohort provides diabetes care to people with type 2 diabetes living in the West-Friesland region in the Netherlands [20]. Medical assessment is performed during patients’ annual visit to the DCS research centre and individuals are invited to participate in the DCS research. We replicated the association of the identified metabolomic biomarkers with incident CVD in HKDR and the DCS cohort; the predictive value of biomarkers for CVD was also assessed in HKDR. A detailed description of the two cohorts and baseline characteristics across the three cohorts are available in electronic supplementary material (ESM) Methods and Results and ESM Tables 1–3.

### Demographic and laboratory measurements

During recruitment, demographic data, medication and medical history were documented via face-to-face interview based on standardised questionnaires. Sex was determined as per self-reported by study participants. BP was measured in both arms after ≥5 min sitting and the mean value was used for analysis. BMI was calculated as weight in kilograms divided by height in metres squared. Blood samples after at least 8 h overnight fasting were measured for HbA_1c_, serum creatinine and lipid profile with certificated routine assays at local laboratories. Albumin was quantified in a random spot urine sample using immunoturbidimetry [22, 23]. Serum creatinine was measured by Jaffe’s kinetic method [22, 23] and eGFR calculated using the Chronic Kidney Disease Epidemiology Collaboration (CKD-EPI) equation.

### Metabolomic profiling

Metabolomic profiles in fasting sera stored (−80°C, fresh from any freeze–thaw cycles) at PWH were quantified using targeted high-throughput NMR spectroscopy (Nightingale Health, Helsinki, Finland). A total of 170 metabolites were quantified simultaneously, including: absolute concentrations of lipoproteins and lipid contents within 14 lipoprotein subclasses, conventional lipids and low-molecular-weight metabolites (LMWMs), including amino acids, ketone bodies, glycolysis-related metabolites and glycoprotein acetyls (GlycA) as well as ratios of fatty acids to total fatty acids. The NMR platform has been extensively applied in large-scale epidemiological studies [18, 25, 26] and experimental details have been published [27]. Measures (phenylalanine, fatty acids, lipid traits, ApoA-1, ApoB, creatinine and albumin) were correlated between NMR and MS or clinical biochemistry [18, 25] and the median CV (IQR) was 5.0% (2.7–6.7%) [25].

Among the 2000 participants profiled from HKDB, 1991 participants were included in the analysis, after excluding two samples failing quality control and seven non-type 2 diabetes participants (ESM Fig. 1). Among the included samples, we assessed the measures compared with clinical biochemistry, including total cholesterol, HDL-cholesterol (HDL-C), LDL-C, triglycerides, fasting glucose and serum creatinine and the Pearson’s correlation coefficients ranged from 0.80 to 0.98 (ESM Fig. 2). ESM Table 4 summarises the measurement quality and distribution of each metabolite among the samples. No measures had ≥20% missing values and were all included in the analysis; zero values, indicating below the detection limit, were imputed with half of the minimum in each measurement.

Samples from HKDR (*N*=93) and the DCS cohort (*N*=1204) were also profiled using the same platform (ESM Methods and Results**)**.

### Outcome

Discharge codes based on ICD-9 (http://www.icd9data.com/2007/Volume1/default.htm) retrieved from electronic medical records were used to define CVD. CVD was defined as the first occurrence of cardiovascular death (ICD-10 [https://icd.who.int/browse10/2019/en]: I00-I99, retrieved from the Hong Kong Death Registry), coronary heart disease (myocardial infarction, ischaemic heart disease, or angina pectoris), stroke (ischaemic stroke except transient ischaemic attack, haemorrhagic stroke, or acute but ill-defined cerebrovascular disease), peripheral vascular disease (amputation, gangrene, or peripheral revascularisation), or hospitalisation for heart failure [21].

### Statistical analysis

Continuous variables were presented as mean ± SD or median (IQR) and differences were compared by *t* test or Wilcoxon rank sum test as appropriate. Categorical variables were presented as number (%) and compared by χ^2^ test. To account for skewed distribution and facilitate interpretation, all metabolites were log_e_-transformed before being standardised to SD. The proportional hazards assumption was tested by scaled Schoenfeld residuals for all variables. False discovery rate (FDR) by the Benjamini–Hochberg procedure <0.05, which is more appropriate for ‘omics’ data, was considered significant to account for multiple testing of intercorrelated metabolites [28]. All analyses were performed in R version 4.0.3 (R Foundation for Statistical Computing, Vienna, Austria). Packages including *survival*, *boot*, *prioritylasso*, *survIDINRI* and *nricens* were used for the analysis.

The cross-sectional associations of metabolites with CKD (baseline eGFR<60 ml/min per 1.73 m^2^) or severely increased albuminuria (urinary albumin/creatinine ratio [UACR] >30 mg/mmol) were separately examined by linear regression, with the metabolite as dependent variable. Two models were considered: unadjusted and adjusted for age, male sex, ever smoking, diabetes duration, systolic BP (SBP), BMI, HbA_1c_, oral glucose-lowering drugs, insulin, antihypertensive drugs, renin–angiotensin-system (RAS) blockers, lipid-lowering drugs, statins, diabetic retinopathy and CVD history. Additionally, severely increased albuminuria (or CKD) was included as a covariate for the analysis of CKD (or severely increased albuminuria).

Among participants without prevalent CVD (*N*=1447), the crude association of CKD or severely increased albuminuria with incident CVD was assessed using Cox proportional hazards model. Given the established association between DKD and incident CVD, metabolites cross-sectionally associated with CKD or severely increased albuminuria were further examined for the prospective associations with incident CVD (*N*=1447) using Cox proportional hazards models, adjusting for the same covariates mentioned above, excluding history of CVD. To identify metabolomic biomarkers independent of conventional risk factors including DKD, metabolites remaining nominally significant (*p*<0.05) after further adjusting for CKD and severely increased albuminuria were assessed for the prognostic value. To account for multicollinearity and data dimensionality, priority least absolute shrinkage and selection operator (priority-Lasso) Cox regression was used to retain metabolites with non-zero coefficients [29]. Two blocks were defined: the first block was unpenalised and included 14 covariates, CKD and severely increased albuminuria; the second block comprised all metabolites associated with incident CVD. The optimal penalisation parameter λ in the second block was determined by the one with minimal cross-validated error as determined by tenfold cross-validation [29]. To account for overfitting, the process was repeated by 1000-times bootstrapping.

To assess the predictive value of the identified metabolomic biomarkers, a risk score comprising the selected metabolites, age and sex was compared with the risk score containing conventional risk factors (original model) and an established prediction model (RECODe model) [30]. The incremental predictive value of the metabolomic biomarkers was also assessed over the original and RECODe models. The predictive value was assessed using C statistic, integrated discrimination improvement (IDI), categorical and continuous net reclassification improvement (NRI). The calculation of IDI and NRI was based on 5-year risk and the risk categories for categorical NRI were <5%, 5–10% and >10%. The 95% CI was estimated by 1000-times bootstrapping. As missing values in each covariate were small (≤2.0%), multiple imputation was not performed. Key R codes are provided in the ESM Methods and Results.

### Sensitivity analyses

We examined cross-sectional associations of metabolites with eGFR and UACR and included eGFR and UACR as covariates in the prospective analysis. We further adjusted for sodium–glucose cotransporter-2 inhibitors (SGLT2i) use during follow-up (*N*=384) in the prospective analysis, as none of the participants were on SGLT2i at baseline. To assess the robustness of variable selection, backward elimination based on Akaike’s information criterion with 1000-times bootstrapping was also performed.

## Results

### Baseline characteristics (*N*=1991)

Table 1 summarises the baseline characteristics of participants. Briefly, mean age was 61.1 years, 59.7% were male, mean diabetes duration was 11.4 years and 27.3% had prevalent CVD. The mean eGFR was 75.8 ml/min per 1.73 m^2^ and 545 participants had prevalent CKD; the median UACR was 2.7 mg/mmol and 399 participants had prevalent severely increased albuminuria. Characteristics of participants with or without CKD or severely increased albuminuria are summarised in Table 1.

**Table 1:** Baseline characteristics of the study population by CKD and severely increased albuminuria status

Variable	Missing (%)	Overall *N*=1991	CKD		*p* value	Macroalbuminuria		*p* value
			No, *N*=1434	Yes, *N*=545		No, *N*=1554	Yes, *N*=399	
Age, years	3 (0.2)	61.1±11.0	58.6±10.6	67.8±8.9	<0.0001	60.3±11.0	64.5±10.3	<0.0001
Male sex	0	1189 (59.7)	837 (58.4)	342 (62.8)	0.085	909 (58.5)	258 (64.7)	0.029
Smoking, ever	1 (0.1)	672 (33.8)	469 (32.7)	197 (36.2)	0.163	505 (32.5)	151 (37.8)	0.050
Diabetes duration, years	10 (0.5)	11.4±8.7	9.7±8.0	15.9±8.7	<0.0001	10.4±8.4	15.3±8.6	<0.0001
SBP, mmHg	7 (0.4)	135.5±18.4	133.0±17.8	141.7±18.4	<0.0001	132.7±17.4	145.8±18.2	<0.0001
BMI, kg/m^2^	8 (0.4)	26.5±4.6	26.3±4.7	26.9±4.4	0.009	26.2±4.5	27.8±4.9	<0.0001
HbA_1c_, mmol/mol	22 (1.1)	58.8±15.6	58.3±15.7	60.1±15.3	0.023	57.9±15.1	62.8±17.0	<0.0001
HbA_1c_, %	22 (1.1)	7.5±1.4	7.5±1.4	7.7±1.4	0.023	7.5±1.4	7.9±1.6	<0.0001
eGFR, ml/min per 1.73 m^2^	12 (0.6)	75.8±26.4	89.4±14.6	40.0±14.1	<0.0001	83.1±21.2	48.5±26.1	<0.0001
G1		748 (37.6)	748 (52.2)	NA		706 (45.4)	35 (8.8)	
G2		686 (34.5)	686 (47.8)	NA		600 (38.6)	82 (20.6)	
G3a		230 (11.6)	NA	230 (42.2)		150 (9.7)	74 (18.6)	
G3b		168 (8.4)	NA	168 (30.8)		76 (4.9)	87 (21.8)	
G4		123 (6.2)	NA	123 (22.6)		17 (1.1)	100 (25.1)	
G5		24 (1.2)	NA	24 (4.4)		3 (0.2)	21 (5.3)	
UACR, mg/mmol	38 (1.9)	2.7 (0.7–17.4)	1.4 (0.6–5.5)	35.2 (4.9–156.9)	<0.0001	1.4 (0.6–4.7)	131.7 (62.9–251.7)	<0.0001
A1		1012 (50.8)	914 (63.7)	97 (17.8)		1012 (65.1)	NA	
A2		542 (27.2)	392 (27.3)	149 (27.3)		542 (34.9)	NA	
A3		399 (20.0)	117 (8.2)	282 (51.7)		NA	399 (100)	
KDIGO risk	40 (2.0)							
Low		914 (45.9)	914 (63.7)	NA		914 (58.8)	NA	
Moderately increased		456 (22.9)	392 (27.3)	64 (11.7)		456 (29.3)	NA	
High		231 (11.6)	117 (8.2)	114 (20.9)		114 (7.3)	117 (29.3)	
Very high		350 (17.6)	NA	350 (64.2)		68 (4.4)	282 (70.7)	
Triglycerides, mmol/l	12 (0.6)	1.3 (1.0–2.0)	1.3 (0.9–1.9)	1.6 (1.1–2.3)	<0.0001	1.3 (0.9–1.8)	1.8 (1.2–2.6)	<0.0001
Total cholesterol, mmol/l	12 (0.6)	4.4±1.0	4.4±0.9	4.3±1.0	0.200	4.3±0.9	4.5±1.2	0.0005
HDL-C, mmol/l	13 (0.7)	1.3±0.4	1.3±0.4	1.2±0.4	<0.0001	1.3±0.4	1.2±0.4	0.0002
LDL-C, mmol/l	26 (1.3)	2.3±0.8	2.4±0.8	2.3±0.8	0.014	2.3±0.7	2.4±0.9	0.291
Diabetic retinopathy	0	517 (26.0)	332 (23.2)	180 (33.0)	<0.0001	354 (22.8)	149 (37.3)	<0.0001
CVD	0	544 (27.3)	304 (21.2)	232 (42.6)	<0.0001	375 (24.1)	147 (36.8)	<0.0001
Oral glucose-lowering drugs	39 (2.0)	1682 (84.5)	1267 (88.4)	408 (74.9)	<0.0001	1362 (87.6)	295 (73.9)	<0.0001
Insulin	26 (1.3)	753 (37.8)	421 (29.4)	323 (59.3)	<0.0001	486 (31.3)	254 (63.7)	<0.0001
Lipid-lowering drugs	15 (0.8)	1360 (68.3)	912 (63.6)	440 (80.7)	<0.0001	1014 (65.3)	320 (80.2)	<0.0001
Statins	7 (0.4)	1333 (67.0)	892 (62.2)	437 (80.2)	<0.0001	999 (64.3)	313 (78.5)	<0.0001
Antihypertensive drugs	19 (1.0)	1524 (76.5)	999 (69.7)	516 (94.7)	<0.0001	1114 (71.7)	380 (95.2)	<0.0001
RAS blockers	7 (0.4)	1183 (59.4)	767 (53.5)	412 (75.6)	<0.0001	853 (54.9)	312 (78.2)	<0.0001

Data are presented as mean ± SD, number (percentage) or median (IQR)

*p* values for differences between CKD/severely increased albuminuria were obtained by *t* test, χ^2^ test, or Wilcoxon rank sum test as appropriate

KDIGO, Kidney Disease: Improving Global Outcomes; NA, not applicable

### Metabolites cross-sectionally associated with DKD (*N*=1991)

**Fig. 1:**
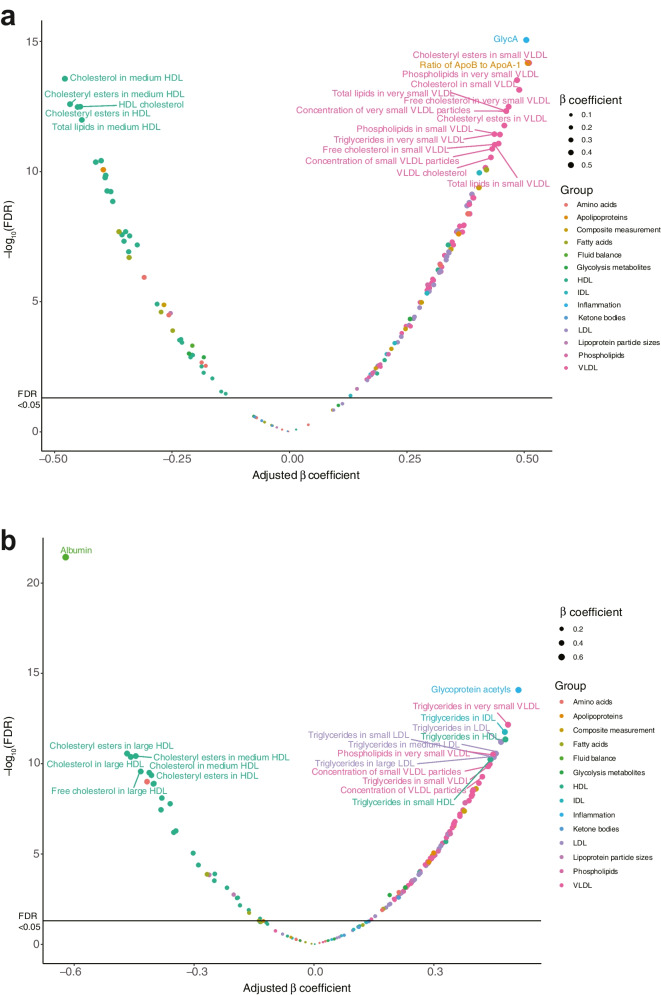
Metabolites associated with CKD (**a**) or severely increased albuminuria (**b**). Estimated by linear regression adjusted for age, male sex, ever smoking, diabetes duration, SBP, BMI, HbA_1c_, oral glucose-lowering drugs, insulin, antihypertensive drugs, lipid-lowering drugs, RAS blockers, statins, diabetic retinopathy and severely increased albuminuria (for the association with severely increased albuminuria, CKD was included instead). Metabolites were log_e_-transformed and scaled to SD. The top 20 most significant metabolites were named

At FDR<0.05, 151 metabolites were associated with CKD (Fig. 1). Cholesterol, phospholipids and total lipids in ApoB-containing lipoproteins were positively, while lipids in HDL were inversely, associated with CKD; concentrations of lipoprotein particles exhibited similar patterns. Triglycerides across all lipoproteins were positively associated with CKD, except triglycerides in large HDL. ApoB and ApoB/ApoA-1 were positively, while ApoA-1 was negatively, associated with CKD. Other lipids, including phosphoglycerides, total cholines, phosphatidylcholines and sphingomyelins, were positively associated with CKD. Relative concentrations of *n*-6 fatty acids, polyunsaturated fatty acids (PUFAs), docosahexaenoic acid (DHA) and PUFAs/monounsaturated fatty acids (MUFAs) were inversely, while relative concentration of MUFAs was positively, associated with CKD. For LMWMs, glycine, phenylalanine, citrate and GlycA were positively, while leucine, valine, tyrosine, glucose, lactate and albumin were negatively, associated with CKD.

At FDR<0.05, 128 metabolites were associated with severely increased albuminuria (Fig. 1). Cholesterol, phospholipids and total lipids in VLDL and LDL were positively, while lipids in larger HDL were negatively, associated with severely increased albuminuria; concentrations of lipoprotein particles exhibited similar patterns. Triglycerides in non-HDL and medium and small HDL were associated with severely increased albuminuria. Other traits including ApoB and ApoB/ApoA-1, MUFAs, isoleucine, phenylalanine, glucose, citrate, 3-hydroxybutyrate, creatinine and GlycA were positively, while PUFAs, DHA, PUFAs/MUFAs, tyrosine and albumin were negatively, associated with severely increased albuminuria.

When eGFR or UACR was assessed as the dependent variable, 148 overlapping metabolites were associated with CKD and 125 overlapping metabolites were associated with severely increased albuminuria at FDR<0.05.

### Associations between DKD-related metabolites and incident CVD (*N*=1447)

Of metabolites associated with CKD (*N*=151) or severely increased albuminuria (*N*=128), 123 metabolites were associated with both CKD and severely increased albuminuria, 28 metabolites only associated with CKD, and five metabolites only associated with severely increased albuminuria. Consequently, 156 metabolites associated with DKD were included in the prospective analysis.

**Fig. 2:**
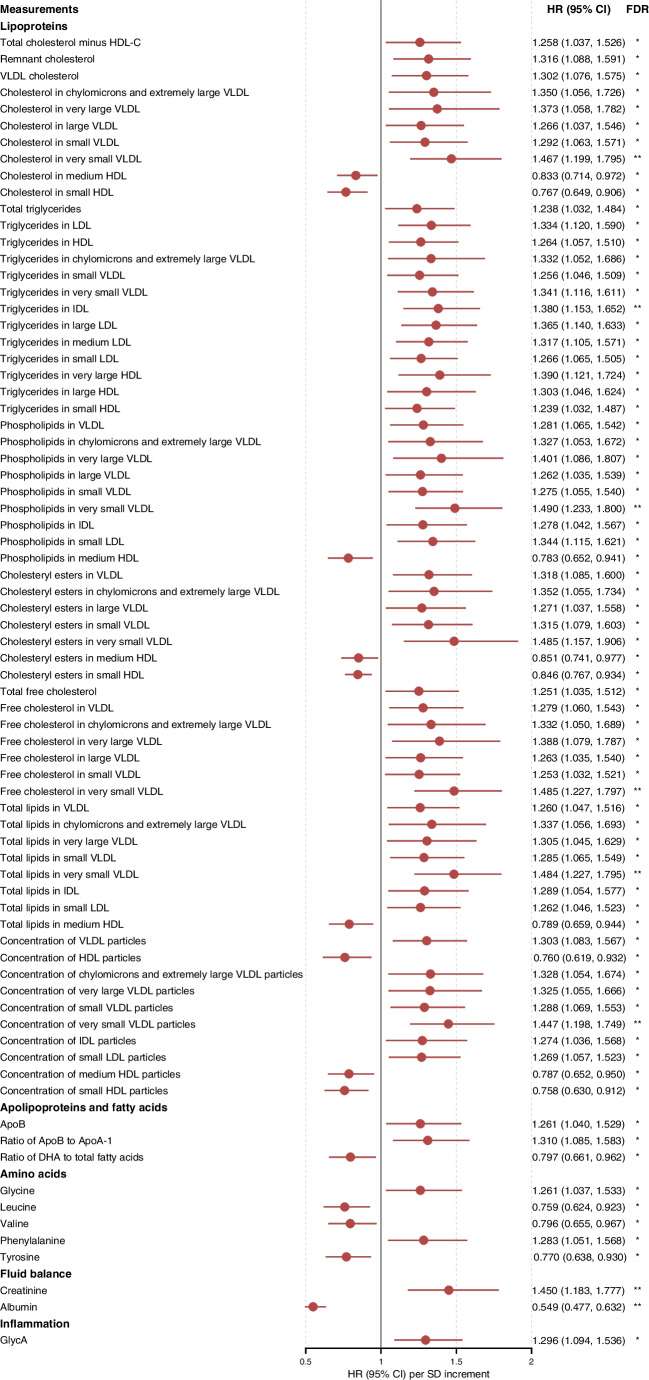
Associations between DKD-related metabolites and incident CVD. Estimated by Cox regression adjusted for age, male sex, ever smoking, diabetes duration, SBP, BMI, HbA_1c_, oral glucose-lowering drugs, insulin, antihypertensive drugs, lipid-lowering drugs, RAS blockers, statins and diabetic retinopathy. Metabolites were log_e_-transformed and scaled to SD. *p<0.05; **p<0.01

Among 1447 participants without prevalent CVD, 125 (8.6%) participants developed CVD over median (IQR) 5.2 (5.0–5.4) years of follow-up, corresponding to an incidence rate (95% CI) of 17.5 (14.6, 20.9) per 1000-person-years. Both CKD and severely increased albuminuria were associated with incident CVD (HR 3.77 [95% CI 2.65, 5.37] and 3.96 [2.77, 5.67] for CKD and severely increased albuminuria, respectively). At FDR<0.05, 116 metabolites were associated with incident CVD in the unadjusted model and 75 metabolites remained significant after accounting for confounders (Fig. 2). Briefly, triglycerides in all lipoproteins were positively associated with incident CVD. Other lipid components in TRLs and LDL were positively, while lipid components in medium and small HDL were inversely, associated with incident CVD with lipoprotein concentrations exhibiting similar patterns. ApoB, ApoB/ApoA-1, glycine, phenylalanine and GlycA were associated with higher risk of CVD, while DHA, leucine, valine, tyrosine and albumin were inversely associated with CVD.

### Prognostic value of selected metabolites for incident CVD (*N*=1447)

Among the 75 metabolites associated with incident CVD, 22 remained nominally significant after further adjusting for CKD and severely increased albuminuria (ESM Fig. 3, ESM Table 5), including concentrations of very small VLDL and small HDL and their lipid components, triglycerides in intermediate-density lipoprotein (IDL), LDL and larger HDL, leucine and albumin. Albumin, triglycerides in large HDL and phospholipids in small LDL were most consistently selected by priority-Lasso (ESM Table 6). The key selection by priority-Lasso was well-represented in backward elimination.

A metabolite score (triglycerides in large HDL plus phospholipids in small LDL minus albumin) was strongly associated with incident CVD (HR 1.43 per SD [95% CI 1.28, 1.59] and 3.31 [1.93, 5.70] for tertile 3 vs tertile 1; ESM Table 7). The three metabolites reached a C statistic (95% CI) of 0.725 (0.672, 0.778) and C statistic of the metabolomic model comprising age, sex and the three metabolites (0.765 [0.718, 0.812]) was comparable with that of traditional risk factors (0.762 [0.717, 0.807], *P*=0.893) and RECODe model (0.765 [0.719, 0.812], *P*=0.994; ESM Fig. 4) [30]. The metabolites further improved CVD risk prediction (improvement in C statistic=0.035 and 0.030, *P*=0.014 and 0.007 over the fully adjusted model and RECODe model, respectively) as well as IDI, continuous NRI and correct reclassification of non-cases for incident CVD (Table 2).

**Table 2:** Predictive utility of the selected metabolites for incident CVD

Variable	Original model^a^	RECODe model^b^
IDI for 5-year risk (95% CI)	0.067 (0.032, 0.110)	0.039 (0.016, 0.070)
Categorical NRI for 5-year risk^c^ (95% CI)		
Case	0.070 (−0.045, 0.152)	−0.013 (−0.060, 0.128)
Non-case	0.075 (0.019, 0.116)	0.065 (0.014, 0.098)
Overall	0.144 (−0.001, 0.245)	0.052 (−0.025, 0.202)
Continuous NRI for 5-year risk (95% CI)		
Case	0.283 (0.079, 0.441)	0.129 (0.017, 0.363)
Non-case	0.258 (0.121, 0.382)	0.102 (−0.031, 0.241)
Overall	0.541 (0.259, 0.759)	0.232 (0.042, 0.536)

Selected metabolites: albumin, triglycerides in large HDL, and phospholipids in small LDL

^a^ Original model: age, male sex, ever smoking, diabetes duration, SBP, BMI, HbA_1c_, oral glucose-lowering drugs, insulin, antihypertensive drugs, lipid-lowering drugs, RAS blockers, statins, diabetic retinopathy, severely increased albuminuria and CKD

^b^ RECODe model: age, sex, ever smoking, SBP, HbA_1c_, total cholesterol, HDL-C, eGFR, log_e_ (UACR), antihypertensive drugs and lipid-lowering drugs

^c^ Risk classification for categorical NRI was: <5%, 5–10% and >10%

95% CI was estimated by 1000-times bootstrapping

The metabolite score was also associated with CVD in HKDR (HR 1.76 per SD [95% CI 1.34, 2.31] and 5.72 [2.24, 14.61] for tertile 3 vs tertile 1; ESM Table 8) and remained significant after adjusting for some confounders (HR 1.74 per SD [95% CI 1.33, 2.28] and 11.12 [3.68, 33.61] for tertile 3 vs tertile 1); further adjusting for CKD and severely increased albuminuria did not change the association. The selected metabolites reached a C statistic of 0.734 (0.621, 0.846) and further improved CVD risk prediction over RECODe model (improvement in C statistic=0.042, *P*=0.043; ESM Fig. 5).

In the DCS cohort, all three metabolites have been associated with impaired kidney function [9]. Over a mean 8.3±3.2 years of follow-up, 141 (11.7%) participants developed incident CVD. The metabolite score was associated with incident CVD (HR 1.16 per SD [95% CI 1.06, 1.27], and 1.99 [1.30, 3.03] for tertile 3 vs tertile 1; ESM Table 9). Adjustment for the same covariates as in the HKDB analysis attenuated the association.

### Sensitivity analyses

Of 75 metabolites associated with incident CVD, 13 remained significant after further adjusting for eGFR and UACR, including two selected metabolites (albumin and triglycerides in large HDL) (ESM Table 10). All metabolites were associated with CVD after accounting for SGLT2i use during follow-up (ESM Table 11).

## Discussion

Applying NMR metabolomics in a well-characterised type 2 diabetes cohort, we comprehensively examined the cross-sectional associations of lipoproteins, lipids and LMWMs with DKD and prospective associations of DKD-related metabolites with incident CVD, identified and assessed metabolomic biomarkers for incident CVD prediction. We found that: (1) TRLs associated with both DKD and incident CVD; (2) HDL inversely associated with DKD and the inverse association with incident CVD appeared mainly driven by smaller (medium and small) HDL; (3) triglycerides across all lipoproteins associated with CVD; and (4) replicated in both Chinese and Europeans, metabolomic biomarkers performed comparably to conventional risk factors and improved CVD risk stratification beyond established prediction models. The results demonstrate profound metabolomic alterations in DKD and close relation with development of CVD, highlighting potential molecular links between DKD and CVD and potential application of metabolomics for diabetes complication prediction.

Some metabolic alterations associated with decreased eGFR are common across different populations and we further identified metabolites associated with severely increased albuminuria in Chinese (ESM Tables 12–13). Consistently, TRLs were associated with decreased eGFR [9, 10] and also with severely increased albuminuria in our study. TRLs have been associated with CVD [31]; larger differences in TRLs in decreased eGFR were found in people with vs without diabetes [10], suggesting a potential role of TRLs for residual CVD risk in people with DKD. In our prospective analysis, TRLs were associated with incident CVD, with VLDL exhibiting the strongest association, although VLDL, IDL and ApoB were all associated with CVD. Hepatic VLDL production and secretion is increased by insulin resistance [32] and altered metabolism of ApoC-3 in CKD further elevates TRLs by overproduction and impaired clearance [6]. All ApoB-containing lipoproteins, including TRLs, can enter the arterial intima leading to cholesterol deposition [7]. In contrast to LDL for which oxidative modification is usually required before phagocytosis, larger TRLs can be trapped more easily and can be directly phagocytised by macrophages to form foam cells [33]. Moreover, hydrolysis of triglycerides in TRLs by lipoprotein lipase can liberate NEFA, inducing inflammation, promoting atherosclerosis [34].

CKD modifies HDL structure and composition, which may partly explain the increased CVD risk in CKD [35]. Consistent with previous findings [9], we found that HDL was negatively associated with CKD and severely increased albuminuria; the association with CKD was stronger. HDL was inversely associated with CVD in our prospective analysis and the association appeared limited to medium and small HDL. However, in previous population-based studies the inverse association between HDL and CVD was limited to large and medium HDL [18, 25]. HDL’s potential modification by diabetes [36] and CKD [35] may partly explain the contrasting results. Furthermore, a recent MR analysis found that medium and small HDL were CVD-protective [37]. Our observed association of small HDL appeared independent of DKD, which is consistent with findings that small HDL has greater atheroprotective capacities via reverse cholesterol transport, anti-inflammatory, antioxidant and endothelial protection [8, 24]. Further studies are warranted to investigate whether detailed HDL composition (proteins, lipids or enzymes) or HDL function may be potential modulators [35]. We replicated previous findings that LDL was associated with DKD [9, 10] and that small LDL was associated with higher CVD risk.

Triglycerides across all lipoproteins were associated with DKD and incident CVD, including TRLs, LDL and HDL. Despite the fact that 67% of participants were on statins, and cholesterol in LDL was not associated with incident CVD, triglycerides in LDL were associated with CVD in our analysis. In people with prediabetes (impaired glucose tolerance and/or impaired fasting glucose) or diabetes and stable coronary artery disease (73.9% on statins), LDL triglycerides were associated with CVD and improved CVD risk prediction, indicating the prognostic value of LDL triglycerides for residual risk [38].

Lower albumin has been associated with DKD [10] and frailty in older people with type 2 diabetes [39] and albumin levels are inversely associated with CVD or mortality in people with CKD [40], suggesting that as a marker linked with malnutrition, liver and kidney dysfunction and inflammation, albumin may partly capture the integrated altered metabolic signature in diabetes and thus associates with adverse outcomes. As a validated marker for systemic inflammation, GlycA was associated with DKD [9, 10] and incident CVD [18, 25], although further adjustment for DKD attenuated the association with CVD. Taken together, our findings suggest that low-grade inflammation in diabetes may be one of the pathogenetic pathways for diabetes complications.

Other lipids, including sphingomyelins, were also associated with DKD [9, 10], however, none were associated with incident CVD, consistent with previous findings that sphingomyelins were associated with DKD but not CVD [41]. In line with previous studies, MUFAs were positively associated with DKD [10], however, PUFAs were negatively linked with DKD in our analysis and the inverse association was mainly driven by DHA. DHA was inversely associated with macrovascular events in the Action in Diabetes and Vascular Disease: Preterax and Diamicron Modified Release Controlled Evaluation (ADVANCE) study [42], which supports our result that DHA as a marker negatively linked with DKD was associated with lower risk of CVD. In CKD, dysfunctional activity of phenylalanine hydroxylase impairs the conversion of phenylalanine to tyrosine [43]. Accordingly, we found that higher phenylalanine and lower tyrosine were associated with DKD [9, 10] and were both associated with risk of CVD. Similar to findings from ADVANCE [11], further adjustment for kidney function attenuated associations with CVD, suggesting that the link between dysregulated phenylalanine or tyrosine and CVD may be mediated by kidney dysfunction. Leucine and isoleucine have been associated with decreased eGFR [9], and branched-chain amino acids (BCAAs) have been negatively associated with CKD in a larger study [10]. We found that leucine and valine were negatively associated with CKD and isoleucine was positively associated with severely increased albuminuria. Leucine and valine were also inversely associated with CVD, in line with the inverse association of leucine and valine with all-cause mortality in ADVANCE [11]. The different associations between BCAAs and DKD across studies might be attributed to participant characteristics, dietary intake, medications or analytical strategies.

Integrating information from gene expression and environmental factors and interacting with the microbiome, metabolites may carry molecular information that is not captured by traditional risk factors [8]. Among metabolites associated with CVD independent of conventional risk factors, three metabolites, albumin, triglycerides in large HDL and phospholipids in small LDL, were identified to be most informative for CVD prediction by machine learning method. The metabolite score comprising these three metabolites was strongly associated with CVD, which was validated independently in both Chinese and European cohorts. The selected metabolites performed comparably to conventional risk factors for CVD prediction and improved risk stratification beyond well-established prediction models, highlighting the prognostic value of metabolomic biomarkers for diabetes complications.

Extending the cross-sectional associations between metabolites and DKD, we found some DKD-related metabolites were associated with incident CVD. We further replicated the association between the identified metabolites and incident CVD in HKDR and the Dutch DCS cohort. Other strengths include the extensively phenotyped data and complete follow-up, well-established metabolomics platform with stringent quality control and consistent results across sensitivity analyses. Nevertheless, there are limitations. Only Chinese individuals were included in the discovery analysis, which might limit generalisability of our findings, however, most metabolites associated with DKD in previous studies were replicated in our study and the selected metabolomic biomarkers were validated in two independent cohorts. Around 70% of participants were on lipid-lowering drugs and we could not account for their potential influence on lipoprotein metabolism, although medication use was accounted for and our findings were consistent with a study in people not on lipid therapies [10]. UACR was based on single measurement and to account for intra-individual variability, we used severely increased albuminuria to define albuminuria. Among metabolites ranked by 1000-times bootstrapping priority-Lasso, an arbitrary cut-off (>70%) was applied to select prognostic metabolomic biomarkers. Although fasting samples were profiled, dietary intake and physical activity that may modulate the metabolome [8] were not captured in our cohort. Given the observational design, residual confounding cannot be ruled out and causal inference is not feasible. Although the study population included slightly more men than women, analyses have been adjusted for the sex of the study participants, and the findings should be applicable to both men and women with diabetes.

In conclusion, DKD is linked with alterations in multiple metabolites, including TRLs, HDL, fatty acids, amino acids, albumin and inflammation. Some DKD-related metabolites (TRLs, smaller HDL, leucine and albumin) are also associated with incident CVD. Metabolomic biomarkers provided comparable predictive utility to traditional risk factors and improved CVD risk stratification over established prediction models. Further investigations on pathophysiology and disease prediction of metabolites are warranted.

### Supplementary Information

Below is the link to the electronic supplementary material.

Supplementary file1 (PDF 2.84 MB)

## Data Availability

The datasets used during the current study are available from the corresponding author (RCWM) upon reasonable request.

## Abbreviations

ADVANCE: Action in Diabetes and Vascular Disease: Preterax and Diamicron Modified Release Controlled Evaluation
ApoA-1: Apolipoprotein A-1
ApoB: Apolipoprotein B
ApoC-3: Apolipoprotein C-3
CKD: Chronic kidney disease
DCS: Hoorn Diabetes Care System
DHA: Docosahexaenoic acid
DKD: Diabetic kidney disease
FDR: False discovery rate
GlycA: Glycoprotein acetyls
HDL-C: HDL-cholesterol
HKDB: Hong Kong Diabetes Biobank
HKDR: Hong Kong Diabetes Register
IDI: Integrated discrimination improvement
IDL: Intermediate-density lipoprotein
LDL-C: LDL-cholesterol
LMWMs: Low-molecular-weight metabolites
MR: Mendelian randomisation
MUFAs: Monounsaturated fatty acids
NRI: Net reclassification improvement
Priority-Lasso: Priority least absolute shrinkage and selection operator
PUFAs: Polyunsaturated fatty acids
PWH: Prince of Wales Hospital
RAS: Renin–angiotensin system
SBP: Systolic BP
SGLT2i: Sodium–glucose cotransporter-2 inhibitors
TRLs: Triglyceride-rich lipoproteins
UACR: Urinary albumin/creatinine ratio
