# Supplementary material for: Circulating metabolomic markers linking diabetic kidney disease and incident cardiovascular disease in type 2 diabetes: analyses from the Hong Kong Diabetes Biobank
Source: Diabetologia. 2024 Feb 27;67(5):837–49. doi: 10.1007/s00125-024-06108-5 (PMC10954952; doi:10.1007/s00125-024-06108-5)
Supplement: Supplementary file 1 — Supplementary file1 (PDF 2.84 MB) [file 125_2024_6108_MOESM1_ESM.pdf]

Electronic supplementary material (ESM)

ESM text (methods and results)....................2

ESM Table 4. Measurement quality and distribution of each metabolite among 1991 samples passing quality control....................9

ESM Table 5. Further adjusting for CKD and severely increased albuminuria for metabolites associated with incident CVD at FDR<0.05 (N=1447)....................14

ESM Table 6. Metabolite selection by 10-fold cross-validation priority-Lasso with 1000 bootstrapping and backward elimination based on AIC with 1000 bootstrapping....................17

ESM Table 7. Association of the metabolite score with incident CVD in HKDB....................19

ESM Table 8. Association of the metabolite score with incident CVD in HKDR (N = 93)....................20

ESM Table 9. Association of the metabolite score with incident CVD in the Hoorn Diabetes Care System Cohort (N=141 incident CVD, 1063 without CVD event)....................21

ESM Table 10. Metabolites associated with incident CVD independent of eGFR and UACR (N=1447)....................22

ESM Table 11. Further adjustment for SGLT2i use during follow-up for metabolites associated with incident CVD (N=1447)....................23

ESM Table 12. Associations of metabolites with CKD across different studies....................26

ESM Table 13. Associations of metabolites with albuminuria across different studies....................31

ESM Fig. 1. Flow chart of study participants....................36

ESM Fig. 2. Correlation coefficients of metabolites measured by NMR and clinical biochemical assay. 37

ESM Fig. 3. Metabolites remaining associated with incident CVD after further adjustment for kidney function. 38

ESM Fig. 4. Receiver operating characteristic curves of metabolomic biomarkers for incident CVD in HKDB....................39

ESM Fig. 5. Receiver operating characteristic curves of metabolomic biomarkers for incident CVD in HKDR....................41

ESM text (methods and results)

The Hong Kong Diabetes Register (HKDR)

The HKDR is a quality-improvement program established in 1995, which is based in Prince of Wales Hospital (PWH), the teaching hospital of the Chinese University of Hong Kong. Through incorporating comprehensive and structured assessment of risk factors and diabetes complications for patients attending the outpatient clinics, HKDR invited and recruited participants for prospective follow-up and biobanking of blood samples for research purpose (Yang XL, et al. Diabetologia. 2006). Since 2000, the program has been adopted by the Hong Kong Hospital Authority to set up a territory-wide diabetes risk assessment program via establishing hospital-based diabetes centers (Chan JCN, et al. Diabetes Care. 2019). Initiated in 2014, the Hong Kong Diabetes Biobank recruited participants from 11 diabetes centers at major public hospitals across Hong Kong using the same enrollment and assessment methods (Jin Q, et al. Am J Kidney Dis. 2022).

Serum samples from 93 participants stored at PWH at -80°C were quantified for the metabolome using targeted high-throughput nuclear magnetic resonance spectroscopy (Nightingale Health, Helsinki, Finland), including 53 male participants involved in a nested case-control study on end-stage kidney disease (ESKD) and 40 random participants recruited from 1996 to 2007. Missing values in the case-control study and random samples were imputed with the minimum in each measurement, separately. The following table summarized the baseline characteristics of participants from HKDR. Over a median (interquartile range) of 16.5 (13.1, 18.4) years of observation, 19 participants developed incident cardiovascular disease (CVD, defined as coronary heart disease, stroke, peripheral vascular disease, or hospitalization for heart failure) in the case-control study. For the random participants, 11 CVD events were recorded over a median of 16.9 (14.4, 20.8) years of follow-up. Consequently, a total of 30 CVD cases were observed over 16.7 years of observation among the all the participants.

ESM Table 1. Baseline characteristics of participants from HKDR.

Variable	Combined participants (N = 93)	Case-control study (N = 53)	Random participants (N = 40)
Age, years	56.39 ± 9.11	53.66 ± 5.34	60 ± 11.58
Male gender	72 (77.42)	53 (100)	19 (47.5)
Smoking, ever	35 (37.63)	23 (43.4)	12 (30)
Diabetes duration, years	6.84 ± 5.3	7.66 ± 5.38	5.75 ± 5.06
SBP, mmHg	140.22 ± 22.13	137.43 ± 22.12	143.91 ± 21.87
BMI, kg/m^2	24.44 ± 3.07	24.66 ± 3.04	24.15 ± 3.11
HbA_{1C}, mmol/mol	58.6 ± 19.7	61.2 ± 20.9	55.1 ± 17.7
HbA_{1C}, %	7.51 ± 1.8	7.75 ± 1.91	7.2 ± 1.62
eGFR, mL/min/1.73 m^2	72.83 ± 24.45	70.47 ± 26.33	75.97 ± 21.65
UACR, mg/mmol	2.7 (1.0-33.8)	14.9 (0.8-137.5)	2.2 (1.2-5.9)
Triglycerides, mmol/L	1.4 (1.0-2.2)	1.4 (1.0-2.1)	1.3 (1.0-2.3)
TC, mmol/L	5.08 ± 1.05	5.25 ± 1.16	4.86 ± 0.86
HDL-C, mmol/L	1.32 ± 0.36	1.31 ± 0.37	1.33 ± 0.33
LDL-C, mmol/L	2.99 ± 0.96	3.16 ± 1.04	2.77 ± 0.82
Diabetic retinopathy	30 (32.26)	21 (39.62)	9 (22.5)
Cardiovascular disease	9 (9.68)	0	9 (22.5)
Oral antihyperglycemic drugs	63 (67.74)	36 (67.92)	27 (67.5)
Insulin	16 (17.2)	11 (20.75)	5 (12.5)
Lipid lowering drugs	23 (24.73)	11 (20.75)	12 (30)

Antihypertensive drugs	47 (50.54)	27 (50.94)	20 (50)
RAS blockers	25 (26.88)	20 (37.74)	5 (12.5)

Data are presented as mean ± SD, number (percentage), or median (interquartile range).

The Hoorn Diabetes Care System (DCS) West-Friesland Cohort

The Hoorn DCS provides diabetes care to people with T2D living in the West-Friesland region in the Netherlands (van der Heijden, et al. BMJ open. 2017). Patients visited the DCS research center annually, during which a medical exam was performed, and blood was drawn for biochemistry. Individuals were advised on health and treatment and have been invited to participate in the DCS research and various rounds of biobanking (N = 5000+).

For the present study, we used two separate samples that were profiled for metabolomic measures (called wave1 and wave 2). Subjects with prevalent CVD were excluded from the present study. Wave 1 included 778 subjects with available plasma samples collected in 2008-2009 and has been described previously ('t Hart LM, et al. J Clin Endocrinol Metab. 2018). The wave 2 sample included 426 subjects and included plasma samples collected between 2008 and 2013. CVD was defined as myocardial infarction, angina pectoris, heart failure, stroke, transient ischaemic attack and PAD, based on self-reported events during the annual visit and verified against the electronic patient registration from the regional hospital and GP. Clinical characteristics are shown in the following table. The distribution of the clinical characteristics and metabolomic measurements were highly similar in both subsets. All samples were stored at -80C after collection and were profiled using the same NMR platform as in the discovery cohort. No significant effect of wave on the results were found (data not shown).

ESM Table 2. Baseline characteristics of participants from the Hoorn DCS cohort.

**Variable**	**Total (N = 1204)**	**Incident CVD (N = 141)**	**No CVD events (N = 1063)**
Age, years	62.4 ± 9.9	65.1 ± 9.5	62.1 ± 9.9 *
Male gender	657 (54)	79 (56)	578 (54)
Smoking, ever	516 (42)	78 (45)	438 (41)
Diabetes duration, years	6.1 ± 4.7	7.2 ± 5.9	6.0 ± 4.5 *
SBP, mmHg	142 ± 19	147 ± 21	142 ± 18 *
BMI, kg/m^2	30.6 ± 5.6	30.1 ± 5.2	30.6 ± 5.7
HbA_{1C}, mmol/mol	49 (43-55)	50 (45-57)	49 (43-55)
HbA_{1C}, %	6.80 ± 0.99	6.96 ± 1.08	6.78 ± 0.97
eGFR, mL/min/1.73 m^2	84 ± 20	79 ± 18	84 ± 20 *
UACR, mg/mmol	0.60 (0.34-1.24)	0.82 (0.44-2.02)	0.60 (0.32-1.15) *
Triglycerides, mmol/L	1.57 (1.14-2.15)	1.63 (1.13-2.25)	1.57 (1.14-2.15)
TC, mmol/L	4.68 ± 1.77	4.73 ± 1.02	4.67 ± 1.85
HDL-C, mmol/L	1.19 ± 0.40	1.18 ±0.34	1.19 ±0.41
LDL-C, mmol/L	2.66 ± 0.89	2.76 ± 0.88	2.65 ± 0.89
Diabetic retinopathy	45 (4)	4 (3)	41 (4)
Cardiovascular disease	-	-	-
Oral antihyperglycemic drugs	978 (81)	107 (76)	871 (82)
Insulin	252 (21)	35 (25)	217 (20)
Lipid lowering drugs	806 (67)	90 (64)	716 (67)

Antihypertensive drugs #	205 (17)	25 (18)	180 (17)
RAS blockers	429 (36)	43 (30)	386 (36%)
Follow-up (years) $	8.3 ± 3.2	3.7 ± 2.8	8.9 ± 2.6

Data are presented as mean ± SD, number (percentage), or median (interquartile range).
* P≤ 0.05 for CVD versus non-CVD; # anti-hypertensive drugs other than RAS blockers;
$ Time till event or last follow-up.

ESM Table 3. Baseline characteristics of participants across the HKDB, HKDR and DCS cohorts.

Variable	HKDB (N = 1991)	HKDR (N = 93)	DCS Cohort (N = 1204)
Age, years	61.1 ± 11.0	56.4 ± 9.1	62.4 ± 9.9
Male gender	1189 (60)	72 (77)	675 (54)
Smoking, ever	672 (34)	35 (38)	516 (42)
Diabetes duration, years	11.4 ± 8.7	6.8 ± 5.3	6.1 ± 4.7
SBP, mmHg	135.5 ± 18.4	140.2 ± 22.1	142.0 ± 19.0
BMI, kg/m^2	26.5 ± 4.6	24.4 ± 3.07	30.6 ± 5.6
HbA_{1C}, mmol/mol	58.8 ± 15.6	58.6 ± 19.7	49 (43-55)
HbA_{1C}, %	7.5 ± 1.4	7.5 ± 1.8	6.80 ± 0.99
eGFR, mL/min/1.73 m^2	75.8 ± 26.4	72.8 ± 24.5	84.0 ± 20.0
UACR, mg/mmol	2.7 (0.7-17.4)	2.7 (1.0-33.8)	0.60 (0.3-1.2)
Triglycerides, mmol/L	1.3 (1.0-2.0)	1.4 (1.0-2.2)	1.57 (1.14-2.15)
TC, mmol/L	4.4 ± 1.0	5.1 ± 1.1	4.7 ± 1.8
HDL-C, mmol/L	1.3 ± 0.4	1.3 ± 0.4	1.2 ± 0.4
LDL-C, mmol/L	2.3 ± 0.8	3.0 ± 1.0	2.7 ± 0.9
Diabetic retinopathy	517 (26)	30 (32)	45 (4)
Cardiovascular disease	544 (27)	9 (10)	-
Oral antihyperglycemic drugs	1682 (85)	63 (68)	978 (81)
Insulin	753 (38)	16 (17)	252 (21)
Lipid lowering drugs	1360 (68)	23 (25)	806 (67)
Antihypertensive drugs	1524 (77)	47 (51)	205 (17)
RAS blockers	1183 (59)	25 (27)	429 (36)

Data are presented as mean ± SD, number (percentage), or median (interquartile range).

R code used for analysis

```
library(survival)
library(dplyr)
library(boot)
library(prioritylasso)
library(survIDINRI)
library(nricens)
library(pROC)

#######################################################################
##### handling of missing values in metabolites
#######################################################################
zero.function<- function(z){
	z<- ifelse(z=="0",min(z[z > 0])/2,z) # replace with half of the minimum in each metabolite
}

#######################################################################
##### linear regression for cross-sectional associations
#######################################################################
lm(meta~ckd,data=dat) # dat: the dataset with both metabolites and clinical variables
lm(meta~ckd+covariates,data=dat)
p.adjust(result$p_value,method="BH") # for FDR correction
# apply loop by introducing only one metabolite in each run and combine the results

#######################################################################
#### cox regression for prospective associations
#######################################################################
# incident CVD
dat<- dat[which(dat$CVE_HIST==FALSE),] # n=1447

# association between CKD/severely increased albuminuria and incident CVD
coxph(Surv(CVE_TIME,CVE_END)~ckd,data=dat)
coxph(Surv(CVE_TIME,CVE_END)~ACR_group,data=dat)

# association between DKD-related metabolites and incident CVD
# read in results on the cross-sectional associations between metabolites and CKD/severely increased albuminuria
# combine metabolites associated with CKD or severely increased albuminuria
coxph(Surv(CVE_TIME,CVE_END)~meta,data=data)
coxph(Surv(CVE_TIME,CVE_END)~meta+covariates,data=data)
p.adjust(result$p_value,method="BH") # for FDR correction
# apply loop by introducing only one metabolite in each run and combine the results

#######################################################################
```

```
#### metabolomic biomarker identification
######################################################################
### further adjustment for CKD and severely increased albuminuria
# read in results on adjusted association between DKD-related metabolites and incident CVD
b<- read.csv("adjusted cox regression for CVE.csv", header=T,stringsAsFactors=F)
FDR_significant<- as.data.frame(subset(b,FDR<0.05))
metabolites<- as.data.frame(FDR_significant$metabolites)

#apply cox regression among the selected metabolites
coxph(Surv(CVE_TIME,CVE_END)~meta+covariates+ckd+macroalbuminuria,data=data_1)
# apply loop by introducing only one selected metabolite in each run and combine the results

# metabolites nominally associated with incident CVD after further adjustment of DKD
b<- as.data.frame(subset(result,raw_p_value<0.05)) # n=22

### priority-Lasso by 1000 times bootstrapping
# define metabolites and clinical covariates
meta_name<- rownames(b) # n=22
cli_name_1<- c("CVD_time","CVD_end", 16 traditional risk factors) # time and status of CVD
and 16 clinical covariates
data_2<- dat %>% select(all_of(cli_name_1),all_of(meta_name))

cli_name_2<- c(16 traditional risk factors) # 16 clinical covariates

# run priority-Lasso model for 1000 times by bootstrapping among the combined dataset
foo<- function(data,incidences){
        d<- data[incidences,]
        x<- d %>% select(all_of(cli_name_2),all_of(meta_name))
        time<- d[,1]
        status<- d[,2]
        y<- Surv(time,status)
        set.seed(1234)
        fit<- prioritylasso(X=x,Y=y,family="cox",type.measure="deviance",
                blocks=list(bp1=1:16,bp2=17:38),
                block1.penalization=FALSE,lamda.type="lamda.min",
                standardize=FALSE,nfolds=10)
        a<- fit$coefficients
        return(a)
}

set.seed(1234)
result<-boot(data_2,foo,R=1000)

# coefficients of Lasso for clinical covariates and metabolites
a<- result$t
```

```
########################################################################
#### predictive utility of the identified metabolomic biomarkers
########################################################################
#### compare the selected metabolites+age+sex with traditional risk factors by C-statistic
cli_name<- c(16 traditional risk factors)
meta_name<- c(3 selected metabolites)

# age + sex + selected metabolites
dat_1<- dat %>% select(all_of(cli_name),all_of(meta_name))
model1<- coxph(Surv(CVE_TIME,CVE_END)~3 selected
metabolites+age+male_sex,data=dat_1)
fit1<- predict(model1,data=dat_1)
roc1<- roc(CVE_END~fit1,data=dat_1)

# traditional risk factors
model2<- coxph(Surv(CVE_TIME,CVE_END)~16 traditional risk factors,data=dat_1)
fit2<- predict(model2,data=dat_1)
roc2<- roc(CVE_END~fit2,data=dat_1)

# DeLong's test for p value
roc.test(roc1,roc2,paired=TRUE)

#### added predictive utility over tratidition risk factors
## C-statistic
# only with metabolites
model1<- coxph(Surv(CVE_TIME,CVE_END)~3 selected metabolites,data=dat)
summary(model1)$concordance

# only traditional risk factors
model2<- coxph(Surv(CVE_TIME,CVE_END)~16 traditional risk factors,data=dat)

# adding metabolites to the model of traditional risk factors
model3<- coxph(Surv(CVE_TIME,CVE_END)~3 selected metabolites+
                    16 traditional risk factors,data=dat)

# apply 1000 bootstrapping to get 95% CI for the difference in C-statistic between model 2 and 3

## IDI
model1<- coxph(Surv(CVE_TIME,CVE_END)~16 traditional risk factors,data=dat_1)
model2<- coxph(Surv(CVE_TIME,CVE_END)~3 selected metabolites+
                    16 traditional risk factors,data=dat_1)
set.seed(123)
IDI.INF(mydata,model1,model2,npert=1000,t0=5)

## NRI
set.seed(123)
```

```
nricens(mdl.std=model1, mdl.new=model2,t0=5,cut=c(0,0.05,0.1,1),niter=1000)
nricens(mdl.std=model1, mdl.new=model2,t0=5,cut=0,niter=1000)
#################################################################
```

ESM Table 4. Measurement quality and distribution of each metabolite among 1991 samples passing quality control.
Zero: concentration below the limit of detection; NA: value was rejected by automatic sample and measurement quality control; TAG: value cannot be quantified due to detected irregularity in sample.

Metabolite	Unit	Zero	NA	TAG	Mean (SD) Before imputation	Mean (SD) After imputation
**Cholesterol**						
Total cholesterol	mmol/L	0 (0%)	0 (0%)	0 (0%)	4.855 (0.9217)	4.855 (0.9217)
Non-HDL-C	mmol/L	0 (0%)	0 (0%)	0 (0%)	3.6158 (0.9325)	3.6158 (0.9325)
Remnant cholesterol	mmol/L	0 (0%)	0 (0%)	0 (0%)	1.6206 (0.4788)	1.6206 (0.4788)
VLDL cholesterol	mmol/L	0 (0%)	0 (0%)	0 (0%)	0.874 (0.3768)	0.874 (0.3768)
LDL cholesterol	mmol/L	0 (0%)	0 (0%)	0 (0%)	1.9953 (0.4715)	1.9953 (0.4715)
HDL cholesterol	mmol/L	0 (0%)	0 (0%)	0 (0%)	1.2391 (0.2879)	1.2391 (0.2879)
Cholesterol in chylomicrons and extremely large VLDL	mmol/L	338 (17%)	0 (0%)	0 (0%)	0.0673 (0.087)	0.0673 (0.087)
Cholesterol in very large VLDL	mmol/L	12 (0.6%)	0 (0%)	0 (0%)	0.0808 (0.0592)	0.0808 (0.0592)
Cholesterol in large VLDL	mmol/L	1 (0.1%)	0 (0%)	0 (0%)	0.1582 (0.1082)	0.1582 (0.1082)
Cholesterol in medium VLDL	mmol/L	8 (0.4%)	0 (0%)	0 (0%)	0.1956 (0.0722)	0.1956 (0.0721)
Cholesterol in small VLDL	mmol/L	0 (0%)	0 (0%)	0 (0%)	0.2008 (0.0711)	0.2008 (0.0711)
Cholesterol in very small VLDL	mmol/L	0 (0%)	0 (0%)	0 (0%)	0.1699 (0.0515)	0.1699 (0.0515)
Cholesterol in IDL	mmol/L	0 (0%)	0 (0%)	0 (0%)	0.7466 (0.1852)	0.7466 (0.1852)
Cholesterol in large LDL	mmol/L	1 (0.1%)	0 (0%)	0 (0%)	1.2114 (0.279)	1.2115 (0.2787)
Cholesterol in medium LDL	mmol/L	1 (0.1%)	0 (0%)	0 (0%)	0.5463 (0.1482)	0.5464 (0.1481)
Cholesterol in small LDL	mmol/L	0 (0%)	0 (0%)	0 (0%)	0.2375 (0.0563)	0.2375 (0.0563)
Cholesterol in very large HDL	mmol/L	61 (3.1%)	0 (0%)	0 (0%)	0.0643 (0.0301)	0.0645 (0.0297)
Cholesterol in large HDL	mmol/L	102 (5.1%)	0 (0%)	0 (0%)	0.2154 (0.1461)	0.2154 (0.1461)
Cholesterol in medium HDL	mmol/L	5 (0.3%)	0 (0%)	0 (0%)	0.4594 (0.121)	0.4595 (0.1206)
Cholesterol in small HDL	mmol/L	0 (0%)	0 (0%)	0 (0%)	0.4998 (0.0599)	0.4998 (0.0599)
**Triglycerides**						
Total triglycerides	mmol/L	0 (0%)	0 (0%)	0 (0%)	1.6297 (1.093)	1.6297 (1.093)
Triglycerides in VLDL	mmol/L	0 (0%)	0 (0%)	0 (0%)	1.251 (0.9862)	1.251 (0.9862)
Triglycerides in LDL	mmol/L	0 (0%)	0 (0%)	0 (0%)	0.1498 (0.0459)	0.1498 (0.0459)
Triglycerides in HDL	mmol/L	0 (0%)	0 (0%)	0 (0%)	0.1295 (0.0536)	0.1295 (0.0536)
Triglycerides in chylomicrons and extremely large VLDL	mmol/L	338 (17%)	0 (0%)	0 (0%)	0.1626 (0.2541)	0.1626 (0.2541)
Triglycerides in very large VLDL	mmol/L	12 (0.6%)	0 (0%)	0 (0%)	0.1591 (0.158)	0.1591 (0.158)
Triglycerides in large VLDL	mmol/L	1 (0.1%)	0 (0%)	0 (0%)	0.2583 (0.2138)	0.2583 (0.2138)
Triglycerides in medium VLDL	mmol/L	8 (0.4%)	0 (0%)	0 (0%)	0.3901 (0.2503)	0.3902 (0.2501)
Triglycerides in small VLDL	mmol/L	0 (0%)	0 (0%)	0 (0%)	0.2037 (0.1077)	0.2037 (0.1077)
Triglycerides in very small VLDL	mmol/L	0 (0%)	0 (0%)	0 (0%)	0.0736 (0.0274)	0.0736 (0.0274)
Triglycerides in IDL	mmol/L	0 (0%)	0 (0%)	0 (0%)	0.0993 (0.0278)	0.0993 (0.0278)

Triglycerides in large LDL	mmol/L	1 (0.1%)	0 (0%)	0 (0%)	0.0984 (0.0269)	0.0984 (0.0269)
Triglycerides in medium LDL	mmol/L	1 (0.1%)	0 (0%)	0 (0%)	0.0349 (0.0123)	0.0349 (0.0123)
Triglycerides in small LDL	mmol/L	0 (0%)	0 (0%)	0 (0%)	0.0165 (0.0078)	0.0165 (0.0078)
Triglycerides in very large HDL	mmol/L	61 (3.1%)	0 (0%)	0 (0%)	0.0063 (0.003)	0.0063 (0.003)
Triglycerides in large HDL	mmol/L	102 (5.1%)	0 (0%)	0 (0%)	0.0199 (0.0109)	0.02 (0.0108)
Triglycerides in medium HDL	mmol/L	5 (0.3%)	0 (0%)	0 (0%)	0.0461 (0.0206)	0.0461 (0.0206)
Triglycerides in small HDL	mmol/L	0 (0%)	0 (0%)	0 (0%)	0.0547 (0.0216)	0.0547 (0.0216)
**Phospholipids**						
Total phospholipids in lipoprotein particles	mmol/L	0 (0%)	0 (0%)	0 (0%)	2.7879 (0.4671)	2.7879 (0.4671)
Phospholipids in VLDL	mmol/L	0 (0%)	0 (0%)	0 (0%)	0.5493 (0.2934)	0.5493 (0.2934)
Phospholipids in LDL	mmol/L	0 (0%)	0 (0%)	0 (0%)	0.6511 (0.1324)	0.6511 (0.1324)
Phospholipids in HDL	mmol/L	0 (0%)	0 (0%)	0 (0%)	1.3248 (0.2735)	1.3248 (0.2735)
Phospholipids in chylomicrons and extremely large VLDL	mmol/L	338 (17%)	0 (0%)	0 (0%)	0.0345 (0.0531)	0.0345 (0.0531)
Phospholipids in very large VLDL	mmol/L	12 (0.6%)	0 (0%)	0 (0%)	0.0514 (0.0484)	0.0514 (0.0484)
Phospholipids in large VLDL	mmol/L	1 (0.1%)	0 (0%)	0 (0%)	0.1014 (0.079)	0.1014 (0.079)
Phospholipids in medium VLDL	mmol/L	8 (0.4%)	0 (0%)	0 (0%)	0.1498 (0.0663)	0.1499 (0.0662)
Phospholipids in small VLDL	mmol/L	0 (0%)	0 (0%)	0 (0%)	0.115 (0.0422)	0.115 (0.0422)
Phospholipids in very small VLDL	mmol/L	0 (0%)	0 (0%)	0 (0%)	0.0962 (0.0305)	0.0962 (0.0305)
Phospholipids in IDL	mmol/L	0 (0%)	0 (0%)	0 (0%)	0.2627 (0.0575)	0.2627 (0.0575)
Phospholipids in large LDL	mmol/L	1 (0.1%)	0 (0%)	0 (0%)	0.3701 (0.074)	0.3701 (0.0738)
Phospholipids in medium LDL	mmol/L	1 (0.1%)	0 (0%)	0 (0%)	0.1829 (0.0425)	0.1829 (0.0425)
Phospholipids in small LDL	mmol/L	0 (0%)	0 (0%)	0 (0%)	0.0981 (0.0198)	0.0981 (0.0198)
Phospholipids in very large HDL	mmol/L	61 (3.1%)	0 (0%)	0 (0%)	0.047 (0.0364)	0.047 (0.0364)
Phospholipids in large HDL	mmol/L	102 (5.1%)	0 (0%)	0 (0%)	0.2158 (0.1308)	0.2163 (0.1301)
Phospholipids in medium HDL	mmol/L	5 (0.3%)	0 (0%)	0 (0%)	0.4154 (0.0931)	0.4156 (0.0922)
Phospholipids in small HDL	mmol/L	0 (0%)	0 (0%)	0 (0%)	0.6451 (0.0921)	0.6451 (0.0921)
**Esterified cholesterol**						
Total esterified cholesterol	mmol/L	0 (0%)	0 (0%)	0 (0%)	3.4262 (0.63)	3.4262 (0.63)
Cholesteryl esters in VLDL	mmol/L	0 (0%)	0 (0%)	0 (0%)	0.4873 (0.1852)	0.4873 (0.1852)
Cholesteryl esters in LDL	mmol/L	0 (0%)	0 (0%)	0 (0%)	1.4545 (0.3675)	1.4545 (0.3675)
Cholesteryl esters in HDL	mmol/L	0 (0%)	0 (0%)	0 (0%)	0.9666 (0.2372)	0.9666 (0.2372)
Cholesteryl esters in chylomicrons and extremely large VLDL	mmol/L	338 (17%)	0 (0%)	0 (0%)	0.0403 (0.0476)	0.0403 (0.0476)
Cholesteryl esters in very large VLDL	mmol/L	12 (0.6%)	0 (0%)	0 (0%)	0.0446 (0.0275)	0.0446 (0.0275)
Cholesteryl esters in large VLDL	mmol/L	1 (0.1%)	0 (0%)	0 (0%)	0.0782 (0.0484)	0.0782 (0.0484)
Cholesteryl esters in medium VLDL	mmol/L	8 (0.4%)	0 (0%)	0 (0%)	0.0919 (0.0377)	0.0919 (0.0377)
Cholesteryl esters in small VLDL	mmol/L	0 (0%)	0 (0%)	0 (0%)	0.1222 (0.0447)	0.1222 (0.0447)
Cholesteryl esters in very small VLDL	mmol/L	0 (0%)	0 (0%)	0 (0%)	0.1093 (0.0354)	0.1093 (0.0354)
Cholesteryl esters in IDL	mmol/L	0 (0%)	0 (0%)	0 (0%)	0.5401 (0.134)	0.5401 (0.134)
Cholesteryl esters in large LDL	mmol/L	1 (0.1%)	0 (0%)	0 (0%)	0.8892 (0.2119)	0.8893 (0.2116)
Cholesteryl esters in medium LDL	mmol/L	1 (0.1%)	0 (0%)	0 (0%)	0.3942 (0.1197)	0.3942 (0.1197)

Cholesteryl esters in small LDL	mmol/L	0 (0%)	0 (0%)	0 (0%)	0.1711 (0.0459)	0.1711 (0.0459)
Cholesteryl esters in very large HDL	mmol/L	61 (3.1%)	0 (0%)	0 (0%)	0.0463 (0.0234)	0.0464 (0.0233)
Cholesteryl esters in large HDL	mmol/L	102 (5.1%)	0 (0%)	0 (0%)	0.1701 (0.1152)	0.1701 (0.1152)
Cholesteryl esters in medium HDL	mmol/L	5 (0.3%)	0 (0%)	0 (0%)	0.3789 (0.1019)	0.379 (0.1018)
Cholesteryl esters in small HDL	mmol/L	0 (0%)	0 (0%)	0 (0%)	0.3711 (0.0476)	0.3711 (0.0476)
**Free cholesterol**						
Total free cholesterol	mmol/L	0 (0%)	0 (0%)	0 (0%)	1.4288 (0.3006)	1.4288 (0.3006)
Free cholesterol in VLDL	mmol/L	0 (0%)	0 (0%)	0 (0%)	0.3867 (0.1995)	0.3867 (0.1995)
Free cholesterol in LDL	mmol/L	0 (0%)	0 (0%)	0 (0%)	0.5407 (0.1221)	0.5407 (0.1221)
Free cholesterol in HDL	mmol/L	0 (0%)	0 (0%)	0 (0%)	0.2726 (0.0553)	0.2726 (0.0553)
Free cholesterol in chylomicrons and extremely large VLDL	mmol/L	338 (17%)	0 (0%)	0 (0%)	0.0269 (0.0397)	0.0269 (0.0397)
Free cholesterol in very large VLDL	mmol/L	12 (0.6%)	0 (0%)	0 (0%)	0.0362 (0.0323)	0.0362 (0.0323)
Free cholesterol in large VLDL	mmol/L	1 (0.1%)	0 (0%)	0 (0%)	0.08 (0.0605)	0.08 (0.0605)
Free cholesterol in medium VLDL	mmol/L	8 (0.4%)	0 (0%)	0 (0%)	0.1036 (0.0437)	0.1036 (0.0436)
Free cholesterol in small VLDL	mmol/L	0 (0%)	0 (0%)	0 (0%)	0.0787 (0.0268)	0.0787 (0.0268)
Free cholesterol in very small VLDL	mmol/L	0 (0%)	0 (0%)	0 (0%)	0.0607 (0.0184)	0.0607 (0.0184)
Free cholesterol in IDL	mmol/L	0 (0%)	0 (0%)	0 (0%)	0.2065 (0.0524)	0.2065 (0.0524)
Free cholesterol in large LDL	mmol/L	1 (0.1%)	0 (0%)	0 (0%)	0.3222 (0.0755)	0.3222 (0.0754)
Free cholesterol in medium LDL	mmol/L	1 (0.1%)	0 (0%)	0 (0%)	0.1521 (0.0346)	0.1521 (0.0346)
Free cholesterol in small LDL	mmol/L	0 (0%)	0 (0%)	0 (0%)	0.0664 (0.0139)	0.0664 (0.0139)
Free cholesterol in very large HDL	mmol/L	61 (3.1%)	0 (0%)	0 (0%)	0.018 (0.0071)	0.018 (0.007)
Free cholesterol in large HDL	mmol/L	102 (5.1%)	0 (0%)	0 (0%)	0.0452 (0.0313)	0.0452 (0.0313)
Free cholesterol in medium HDL	mmol/L	5 (0.3%)	0 (0%)	0 (0%)	0.0805 (0.0206)	0.0805 (0.0205)
Free cholesterol in small HDL	mmol/L	0 (0%)	0 (0%)	0 (0%)	0.1286 (0.0168)	0.1286 (0.0168)
**Total lipids**						
Total lipids in lipoprotein particles	mmol/L	0 (0%)	0 (0%)	0 (0%)	9.2725 (2.1378)	9.2725 (2.1378)
Total lipids in VLDL	mmol/L	0 (0%)	0 (0%)	0 (0%)	2.6742 (1.6185)	2.6742 (1.6185)
Total lipids in LDL	mmol/L	0 (0%)	0 (0%)	0 (0%)	2.7962 (0.6334)	2.7962 (0.6334)
Total lipids in HDL	mmol/L	0 (0%)	0 (0%)	0 (0%)	2.6935 (0.5436)	2.6935 (0.5436)
Total lipids in chylomicrons and extremely large VLDL	mmol/L	338 (17%)	0 (0%)	0 (0%)	0.2643 (0.3933)	0.2643 (0.3932)
Total lipids in very large VLDL	mmol/L	12 (0.6%)	0 (0%)	0 (0%)	0.2913 (0.2643)	0.2913 (0.2643)
Total lipids in large VLDL	mmol/L	1 (0.1%)	0 (0%)	0 (0%)	0.5178 (0.3987)	0.5179 (0.3986)
Total lipids in medium VLDL	mmol/L	8 (0.4%)	0 (0%)	0 (0%)	0.7355 (0.363)	0.7357 (0.3625)
Total lipids in small VLDL	mmol/L	0 (0%)	0 (0%)	0 (0%)	0.5195 (0.2102)	0.5195 (0.2102)
Total lipids in very small VLDL	mmol/L	0 (0%)	0 (0%)	0 (0%)	0.3397 (0.0989)	0.3397 (0.0989)
Total lipids in IDL	mmol/L	0 (0%)	0 (0%)	0 (0%)	1.1086 (0.2538)	1.1086 (0.2538)
Total lipids in large LDL	mmol/L	1 (0.1%)	0 (0%)	0 (0%)	1.6798 (0.3685)	1.68 (0.3679)
Total lipids in medium LDL	mmol/L	1 (0.1%)	0 (0%)	0 (0%)	0.7641 (0.1998)	0.7641 (0.1996)
Total lipids in small LDL	mmol/L	0 (0%)	0 (0%)	0 (0%)	0.3522 (0.0814)	0.3522 (0.0814)
Total lipids in very large HDL	mmol/L	61 (3.1%)	0 (0%)	0 (0%)	0.1176 (0.0662)	0.1179 (0.0657)

Total lipids in large HDL	mmol/L	102 (5.1%)	0 (0%)	0 (0%)	0.4511 (0.2799)	0.4523 (0.278)
Total lipids in medium HDL	mmol/L	5 (0.3%)	0 (0%)	0 (0%)	0.9209 (0.2093)	0.9214 (0.2075)
Total lipids in small HDL	mmol/L	0 (0%)	0 (0%)	0 (0%)	1.1996 (0.1566)	1.1996 (0.1566)
**Concentration of lipoprotein particle**						
Total concentration of lipoprotein particles	mmol/L	0 (0%)	0 (0%)	0 (0%)	0.0234 (0.0032)	0.0234 (0.0032)
Concentration of VLDL particles	mmol/L	0 (0%)	0 (0%)	0 (0%)	2e-04 (1e-04)	2e-04 (1e-04)
Concentration of LDL particles	mmol/L	0 (0%)	0 (0%)	0 (0%)	0.0013 (4e-04)	0.0013 (4e-04)
Concentration of HDL particles	mmol/L	0 (0%)	0 (0%)	0 (0%)	0.0216 (0.0031)	0.0216 (0.0031)
Concentration of chylomicrons and extremely large VLDL particles	mmol/L	338 (17%)	0 (0%)	0 (0%)	0 (0)	0 (0)
Concentration of very large VLDL particles	mmol/L	12 (0.6%)	0 (0%)	0 (0%)	0 (0)	0 (0)
Concentration of large VLDL particles	mmol/L	1 (0.1%)	0 (0%)	0 (0%)	0 (0)	0 (0)
Concentration of medium VLDL particles	mmol/L	8 (0.4%)	0 (0%)	0 (0%)	1e-04 (0)	1e-04 (0)
Concentration of small VLDL particles	mmol/L	0 (0%)	0 (0%)	0 (0%)	1e-04 (0)	1e-04 (0)
Concentration of very small VLDL particles	mmol/L	0 (0%)	0 (0%)	0 (0%)	1e-04 (0)	1e-04 (0)
Concentration of IDL particles	mmol/L	0 (0%)	0 (0%)	0 (0%)	3e-04 (1e-04)	3e-04 (1e-04)
Concentration of large LDL particles	mmol/L	1 (0.1%)	0 (0%)	0 (0%)	7e-04 (2e-04)	7e-04 (2e-04)
Concentration of medium LDL particles	mmol/L	1 (0.1%)	0 (0%)	0 (0%)	4e-04 (1e-04)	4e-04 (1e-04)
Concentration of small LDL particles	mmol/L	0 (0%)	0 (0%)	0 (0%)	2e-04 (1e-04)	2e-04 (1e-04)
Concentration of very large HDL particles	mmol/L	61 (3.1%)	0 (0%)	0 (0%)	3e-04 (1e-04)	3e-04 (1e-04)
Concentration of large HDL particles	mmol/L	102 (5.1%)	0 (0%)	0 (0%)	0.0014 (9e-04)	0.0014 (9e-04)
Concentration of medium HDL particles	mmol/L	5 (0.3%)	0 (0%)	0 (0%)	0.0049 (0.0012)	0.0049 (0.0012)
Concentration of small HDL particles	mmol/L	0 (0%)	0 (0%)	0 (0%)	0.015 (0.0019)	0.015 (0.0019)
**Average diameter of lipoprotein particles**						
Average diameter for VLDL particles	nm	0 (0%)	0 (0%)	0 (0%)	39.4469 (1.6734)	39.4469 (1.6734)
Average diameter for LDL particles	nm	0 (0%)	0 (0%)	0 (0%)	23.5862 (0.1271)	23.5862 (0.1271)
Average diameter for HDL particles	nm	0 (0%)	0 (0%)	0 (0%)	9.48 (0.1911)	9.48 (0.1911)
**Other lipids**						
Phosphoglycerides	mmol/L	0 (0%)	10 (0.5%)	0 (0%)	2.48 (0.5101)	2.48 (0.5101)
Ratio of triglycerides to phosphoglycerides	ratio	0 (0%)	10 (0.5%)	0 (0%)	0.62 (0.2802)	0.62 (0.2802)
Total cholines	mmol/L	0 (0%)	7 (0.4%)	0 (0%)	2.7894 (0.5019)	2.7894 (0.5019)
Phosphatidylcholines	mmol/L	0 (0%)	7 (0.4%)	0 (0%)	2.2553 (0.487)	2.2553 (0.487)
Sphingomyelins	mmol/L	0 (0%)	7 (0.4%)	0 (0%)	0.4786 (0.0809)	0.4786 (0.0809)
**Apolipoproteins**						
Apolipoprotein B	g/L	0 (0%)	0 (0%)	0 (0%)	0.9552 (0.262)	0.9552 (0.262)
Apolipoprotein A1	g/L	0 (0%)	0 (0%)	0 (0%)	1.2204 (0.2322)	1.2204 (0.2322)
Ratio of apolipoprotein B to apolipoprotein A1	ratio	0 (0%)	0 (0%)	0 (0%)	0.8138 (0.2921)	0.8138 (0.2921)
**Fatty acid ratios**						
Ratio of omega-3 FAs to total FAs	%	0 (0%)	22 (1.1%)	0 (0%)	4.8432 (1.3182)	4.8432 (1.3182)
Ratio of omega-6 FAs to total FAs	%	0 (0%)	22 (1.1%)	0 (0%)	36.5245 (2.9794)	36.5245 (2.9794)
Ratio of polyunsaturated FAs to total FAs	%	0 (0%)	22 (1.1%)	0 (0%)	41.3678 (2.8673)	41.3678 (2.8673)

Ratio of monounsaturated FAs to total FAs	%	0 (0%)	22 (1.1%)	0 (0%)	26.6249 (2.1464)	26.6249 (2.1464)
Ratio of saturated FAs to total FAs	%	0 (0%)	22 (1.1%)	0 (0%)	32.0074 (1.4313)	32.0074 (1.4313)
Ratio of linoleic acid to total FAs	%	0 (0%)	22 (1.1%)	0 (0%)	31.4124 (3.1338)	31.4124 (3.1338)
Ratio of docosahexaenoic acid to total FAs	%	0 (0%)	22 (1.1%)	0 (0%)	3.7425 (0.7746)	3.7425 (0.7746)
Ratio of polyunsaturated FAs to monounsaturated FAs	ratio	0 (0%)	22 (1.1%)	0 (0%)	1.5712 (0.2247)	1.5712 (0.2247)
Ratio of omega-6 FAs to omega-3 FAs	ratio	0 (0%)	6 (0.3%)	0 (0%)	8.0809 (2.288)	8.0809 (2.288)
**Amino acids**						
Alanine	mmol/L	0 (0%)	0 (0%)	0 (0%)	0.4788 (0.0973)	0.4788 (0.0973)
Glutamine	mmol/L	0 (0%)	0 (0%)	0 (0%)	0.7395 (0.0886)	0.7395 (0.0886)
Glycine	mmol/L	0 (0%)	4 (0.2%)	0 (0%)	0.2667 (0.0459)	0.2667 (0.0459)
Histidine	mmol/L	0 (0%)	1 (0.1%)	0 (0%)	0.0742 (0.0123)	0.0742 (0.0123)
Total concentration of BCAAs	mmol/L	0 (0%)	0 (0%)	0 (0%)	0.5137 (0.0963)	0.5137 (0.0963)
Isoleucine	mmol/L	0 (0%)	0 (0%)	0 (0%)	0.0796 (0.021)	0.0796 (0.021)
Leucine	mmol/L	0 (0%)	0 (0%)	0 (0%)	0.1478 (0.0314)	0.1478 (0.0314)
Valine	mmol/L	0 (0%)	0 (0%)	0 (0%)	0.2864 (0.0484)	0.2864 (0.0484)
Phenylalanine	mmol/L	0 (0%)	2 (0.1%)	0 (0%)	0.0863 (0.0143)	0.0863 (0.0143)
Tyrosine	mmol/L	0 (0%)	1 (0.1%)	0 (0%)	0.0653 (0.0127)	0.0653 (0.0127)
**Glycolysis related metabolites**						
Glucose	mmol/L	0 (0%)	1 (0.1%)	0 (0%)	7.5742 (2.9651)	7.5742 (2.9651)
Lactate	mmol/L	0 (0%)	2 (0.1%)	0 (0%)	2.5089 (0.6617)	2.5089 (0.6617)
Pyruvate	mmol/L	0 (0%)	2 (0.1%)	0 (0%)	0.0492 (0.0266)	0.0492 (0.0266)
Citrate	mmol/L	0 (0%)	0 (0%)	0 (0%)	0.0783 (0.014)	0.0783 (0.014)
Glycerol	mmol/L	1 (0.1%)	0 (0%)	9 (0.5%)	0.0983 (0.0372)	0.0983 (0.0371)
**Ketone bodies**						
3-Hydroxybutyrate	mmol/L	11 (0.6%)	0 (0%)	4 (0.2%)	0.0896 (0.221)	0.0901 (0.2215)
Acetate	mmol/L	0 (0%)	0 (0%)	0 (0%)	0.0333 (0.0539)	0.0333 (0.0539)
Acetoacetate	mmol/L	0 (0%)	0 (0%)	0 (0%)	0.0429 (0.0786)	0.0429 (0.0786)
Acetone	mmol/L	0 (0%)	1 (0.1%)	0 (0%)	0.0205 (0.0334)	0.0205 (0.0334)
**Fluid balance**						
Creatinine	µmol/L	0 (0%)	0 (0%)	6 (0.3%)	95.4094 (55.1139)	95.4094 (55.1139)
Albumin	g/L	0 (0%)	0 (0%)	0 (0%)	40.5232 (3.7154)	40.5232 (3.7154)
**Inflammation**						
Glycoprotein acetyls	mmol/L	0 (0%)	0 (0%)	0 (0%)	1.0886 (0.1991)	1.0886 (0.1991)

ESM Table 5. Further adjusting for CKD and severely increased albuminuria for metabolites associated with incident CVD at FDR<0.05 (N=1447).

Metabolite	HR (95% CI)	Raw *P* value
Non-HDL-C	1.152 (0.948, 1.4)	0.1543
Remnant cholesterol	1.192 (0.98, 1.45)	0.0781
VLDL cholesterol	1.181 (0.967, 1.443)	0.1026
Cholesterol in chylomicrons and extremely large VLDL	1.293 (1.002, 1.668)	0.048
Cholesterol in very large VLDL	1.237 (0.947, 1.615)	0.1186
Cholesterol in large VLDL	1.163 (0.944, 1.433)	0.1571
Cholesterol in small VLDL	1.159 (0.947, 1.418)	0.1534
Cholesterol in very small VLDL	1.325 (1.084, 1.62)	0.006
Cholesterol in medium HDL	0.935 (0.79, 1.106)	0.4335
Cholesterol in small HDL	0.815 (0.685, 0.971)	0.0217
Total triglycerides	1.149 (0.948, 1.392)	0.1582
Triglycerides in LDL	1.226 (1.016, 1.48)	0.0335
Triglycerides in HDL	1.159 (0.957, 1.405)	0.1315
Triglycerides in chylomicrons and extremely large VLDL	1.283 (1.003, 1.641)	0.0471
Triglycerides in small VLDL	1.151 (0.947, 1.399)	0.1565
Triglycerides in very small VLDL	1.215 (0.997, 1.482)	0.054
Triglycerides in IDL	1.263 (1.042, 1.53)	0.0174
Triglycerides in large LDL	1.253 (1.035, 1.517)	0.0209
Triglycerides in medium LDL	1.209 (1, 1.461)	0.0496
Triglycerides in small LDL	1.166 (0.968, 1.405)	0.1055
Triglycerides in very large HDL	1.301 (1.058, 1.599)	0.0127
Triglycerides in large HDL	1.305 (1.063, 1.602)	0.0108
Triglycerides in small HDL	1.135 (0.933, 1.381)	0.2054
Phospholipids in VLDL	1.172 (0.963, 1.427)	0.1128
Phospholipids in chylomicrons and extremely large VLDL	1.263 (0.993, 1.606)	0.0576
Phospholipids in very large VLDL	1.282 (0.986, 1.667)	0.0634
Phospholipids in large VLDL	1.167 (0.947, 1.437)	0.1466
Phospholipids in small VLDL	1.15 (0.944, 1.401)	0.1648
Phospholipids in very small VLDL	1.337 (1.094, 1.634)	0.0045
Phospholipids in IDL	1.209 (0.994, 1.47)	0.0573
Phospholipids in small LDL	1.227 (1.014, 1.485)	0.0353
Phospholipids in medium HDL	0.858 (0.711, 1.034)	0.1081

Cholesteryl esters in VLDL	1.188 (0.971, 1.453)	0.095
Cholesteryl esters in chylomicrons and extremely large VLDL	1.299 (1.004, 1.681)	0.0467
Cholesteryl esters in large VLDL	1.159 (0.937, 1.433)	0.1746
Cholesteryl esters in small VLDL	1.175 (0.957, 1.442)	0.1238
Cholesteryl esters in very small VLDL	1.341 (1.058, 1.698)	0.0152
Cholesteryl esters in medium HDL	0.946 (0.81, 1.106)	0.4868
Cholesteryl esters in small HDL	0.89 (0.799, 0.99)	0.0327
Total free cholesterol	1.156 (0.955, 1.4)	0.1379
Free cholesterol in VLDL	1.169 (0.959, 1.425)	0.1218
Free cholesterol in chylomicrons and extremely large VLDL	1.27 (0.992, 1.626)	0.0578
Free cholesterol in very large VLDL	1.266 (0.976, 1.642)	0.0759
Free cholesterol in large VLDL	1.166 (0.946, 1.435)	0.1493
Free cholesterol in small VLDL	1.132 (0.928, 1.381)	0.22
Free cholesterol in very small VLDL	1.332 (1.092, 1.625)	0.0047
Total lipids in VLDL	1.16 (0.954, 1.411)	0.1364
Total lipids in chylomicrons and extremely large VLDL	1.279 (1.001, 1.635)	0.0488
Total lipids in very large VLDL	1.208 (0.96, 1.52)	0.1076
Total lipids in small VLDL	1.162 (0.954, 1.417)	0.1362
Total lipids in very small VLDL	1.333 (1.092, 1.627)	0.0046
Total lipids in IDL	1.206 (0.991, 1.468)	0.061
Total lipids in small LDL	1.16 (0.958, 1.404)	0.1292
Total lipids in medium HDL	0.878 (0.727, 1.06)	0.1758
Concentration of VLDL particles	1.184 (0.974, 1.44)	0.0901
Concentration of HDL particles	0.828 (0.671, 1.022)	0.0788
Concentration of chylomicrons and extremely large VLDL particles	1.263 (0.993, 1.606)	0.0571
Concentration of very large VLDL particles	1.221 (0.963, 1.547)	0.099
Concentration of small VLDL particles	1.166 (0.957, 1.421)	0.1285
Concentration of very small VLDL particles	1.303 (1.069, 1.588)	0.0089
Concentration of IDL particles	1.177 (0.961, 1.441)	0.1149
Concentration of small LDL particles	1.165 (0.965, 1.407)	0.1117
Concentration of medium HDL particles	0.868 (0.715, 1.054)	0.1536
Concentration of small HDL particles	0.796 (0.657, 0.964)	0.0199
Apolipoprotein B	1.154 (0.95, 1.402)	0.15
Ratio of apolipoprotein B to apolipoprotein A1	1.177 (0.969, 1.429)	0.1012
Ratio of docosahexaenoic acid to total FAs	0.853 (0.703, 1.034)	0.1055
Glycine	1.168 (0.958, 1.425)	0.1252

Leucine	0.81 (0.663, 0.99)	0.04
Valine	0.861 (0.705, 1.053)	0.1453
Phenylalanine	1.195 (0.969, 1.475)	0.0956
Tyrosine	0.879 (0.718, 1.076)	0.2118
Creatinine	1.076 (0.778, 1.487)	0.6589
Albumin	0.583 (0.495, 0.687)	<0.0001
Glycoprotein acetyls	1.175 (0.972, 1.421)	0.0959

Estimated by Cox regression.

Adjusted for age, male sex, ever smoking, diabetes duration, systolic blood pressure, body mass index, glycated hemoglobin, oral antihyperglycemic drugs, insulin, antihypertensive drugs, lipid-lowering drugs, renin-angiotensin system blockers, statins, diabetic retinopathy, and SGLT2i use during follow-up.

Metabolites were $\log_e$-transformed and scaled to standard deviation.

ESM Table 6. Metabolite selection by 10-fold cross-validation priority-Lasso with 1000 bootstrapping and backward elimination based on AIC with 1000 bootstrapping.

Priority-Lasso		Backward elimination	
**Metabolite**	**N (%)**	**Metabolite**	**N (%)**
**Albumin**	**998 (99.8%)**	**Albumin**	**1000 (100%)**
**Triglycerides in large HDL**	**911 (91.1%)**	Cholesterol in small HDL	778 (77.8%)
**Phospholipids in small LDL**	**730 (73.0%)**	Cholesteryl esters in small HDL	767 (76.7%)
Triglycerides in chylomicrons and extremely large VLDL	654 (65.4%)	Phospholipids in very small VLDL	704 (70.4%)
Leucine	651 (65.1%)	**Triglycerides in large HDL**	**656 (65.6%)**
Triglycerides in very large HDL	602 (60.2%)	Triglycerides in IDL	617 (61.7%)
Cholesteryl esters in very small VLDL	593 (59.3%)	**Phospholipids in small LDL**	**572 (57.2%)**
Concentration of small HDL particles	504 (50.4%)	Cholesteryl esters in very small VLDL	567 (56.7%)
Phospholipids in very small VLDL	490 (49.0%)	Triglycerides in LDL	556 (55.6%)
Cholesteryl esters in small HDL	455 (45.5%)	Triglycerides in large LDL	508 (50.8%)
Cholesteryl esters in chylomicrons and extremely large VLDL	389 (38.9%)	Triglycerides in chylomicrons and extremely large VLDL	503 (50.3%)
Triglycerides in large LDL	350 (35.0%)	Cholesterol in very small VLDL	497 (49.7%)
Cholesterol in small HDL	268 (26.8%)	Free cholesterol in very small VLDL	484 (48.4%)
Triglycerides in medium LDL	263 (26.3%)	Total lipids in very small VLDL	483 (48.3%)
Triglycerides in IDL	225 (22.5%)	Triglycerides in medium LDL	468 (46.8%)
Concentration of very small VLDL particles	220 (22.0%)	Concentration of small HDL particles	438 (43.8%)
Free cholesterol in very small VLDL	189 (18.9%)	Total lipids in chylomicrons and extremely large VLDL	430 (43.0%)
Cholesterol in chylomicrons and extremely large VLDL	170 (17.0%)	Cholesterol in chylomicrons and extremely large VLDL	427 (42.7%)
Cholesterol in very small VLDL	164 (16.4%)	Cholesteryl esters in chylomicrons and extremely large VLDL	394 (39.4%)
Total lipids in chylomicrons and extremely large VLDL	139 (13.9%)	Concentration of very small VLDL particles	314 (31.4%)
Total lipids in very small VLDL	133 (13.3%)	Leucine	306 (30.6%)
Triglycerides in LDL	117 (11.7%)	Triglycerides in very large HDL	289 (28.9%)

Metabolites were $\log_e$-transformed and scaled to standard deviation.

Priority-Lasso: First block included age, male sex, ever smoking, diabetes duration, systolic blood pressure, body mass index, glycated hemoglobin, oral antihyperglycemic drugs, insulin, antihypertensive drugs, lipid-lowering drugs, renin-angiotensin system blockers, statins, diabetic retinopathy, chronic kidney disease, and severely increased albuminuria; second block included 22 metabolites associated with CVD. One thousand iterations of L1-regularized Cox regression with the optimal degree of regularization determined by cross-validated error via 10-fold cross-validation. Metabolites had nonzero coefficients were selected in each iteration.

Backward elimination: all 22 metabolites associated with CVD were included in the Cox regression adjusted for age, male sex, ever smoking, diabetes duration, systolic blood pressure, body mass index, glycated hemoglobin, oral antihyperglycemic drugs, insulin,

antihypertensive drugs, lipid-lowering drugs, renin-angiotensin system blockers, statins, diabetic retinopathy, chronic kidney disease, and severely increased albuminuria. Metabolites with minimum Akaike information criterion were selected in each iteration and the process was repeated for 1000 times.
Priority-Lasso: priority least absolute shrinkage and selection operator Cox regression; AIC: Akaike information criterion.

ESM Table 7. Association of the metabolite score with incident CVD in HKDB.

	**Unadjusted HR (95% CI)**	***P* value**	**Adjusted HR (95% CI)**	***P* value**
Per SD	1.563 (1.436, 1.702)	8.72E-25	1.425 (1.278, 1.589)	1.96E-10
Tertile 1	Reference		Reference	
Tertile 2	1.552 (0.87, 2.767)	0.137	1.515 (0.823, 2.789)	0.183
Tertile 3	4.432 (2.683, 7.324)	6.20E-09	3.312 (1.925, 5.699)	1.52E-05

The metabolites score was calculated as triglycerides in large HDL plus phospholipids in small LDL minus albumin.

Estimated by Cox regression.

Adjusted for age, male sex, ever smoking, diabetes duration, systolic blood pressure, body mass index, glycated hemoglobin, oral antihyperglycemic drugs, insulin, antihypertensive drugs, lipid-lowering drugs, renin-angiotensin system blockers, statins, diabetic retinopathy, chronic kidney disease, and severely increased albuminuria.

Metabolites were $\log_e$-transformed and scaled to standard deviation.

ESM Table 8. Association of the metabolite score with incident CVD in HKDR (N = 93).

	**Model 1**	***P* value**	**Model 2**	***P* value**	**Model 3**	***P* value**	**Model 4**	***P* value**	**Model 5**	***P* value**
Per SD	1.76 (1.34, 2.31)	4.19E-05	1.74 (1.33, 2.28)	5.62E-05	1.67 (1.27, 2.20)	2.45E-04	1.44 (1.07, 1.93)	0.015	1.44 (1.07, 1.94)	0.015
Tertile 1	Reference		Reference		Reference		Reference		Reference	
Tertile 2	1.09 (0.35, 3.38)	0.882	1.58 (0.47, 5.29)	0.462	1.58 (0.47, 5.30)	0.456	1.30 (0.37, 4.52)	0.681	1.17 (0.32, 4.26)	0.817
Tertile 3	5.72 (2.24, 14.61)	2.67E-04	11.12 (3.68, 33.61)	1.98E-05	10.30 (3.38, 31.36)	4.07E-05	6.48 (1.99, 21.15)	0.002	6.33 (1.94, 20.64)	0.002

The metabolites score was calculated as triglycerides in large HDL plus phospholipids in small LDL minus albumin.
Data are expressed as HR (95% CI), estimated by Cox regression.
Metabolites were $\log_e$-transformed and scaled to standard deviation.
Model 1: unadjusted;
Model 2: age, male sex, ever smoking, diabetes duration, systolic blood pressure, body mass index, and glycated hemoglobin;
Model 3: model 2 plus chronic kidney disease;
Model 4: model 2 plus severely increased albuminuria;
Model 5: Model 2 plus chronic kidney disease and severely increased albuminuria.

ESM Table 9. Association of the metabolite score with incident CVD in the Hoorn Diabetes Care System Cohort (N=141 incident CVD, 1063 without CVD event).

	**Unadjusted HR (95% CI)**	***P* value**	**Adjusted HR (95% CI)**	***P* value**
Per SD	1.16 (1.061, 1.268)	0.001	1.083 (0.970, 1.209)	0.156
Tertile 1	Reference		Reference	
Tertile 2	1.442 (0.927, 2.242)	0.104	1.183 (0.733, 1.911)	0.491
Tertile 3	1.985 (1.303, 3.025)	0.001	1.575 (0.958, 2.587)	0.073

The metabolites score was calculated as triglycerides in large HDL plus phospholipids in small LDL minus albumin.

Estimated by Cox regression.

Adjusted for age, male sex, ever smoking, diabetes duration, systolic blood pressure, body mass index, glycated hemoglobin, oral antihyperglycemic drugs, insulin, antihypertensive drugs, lipid-lowering drugs, renin-angiotensin system blockers, statins, diabetic retinopathy, chronic kidney disease, and severely increased albuminuria.

Metabolites were $\log_e$-transformed and scaled to standard deviation.

ESM Table 10. Metabolites associated with incident CVD independent of eGFR and UACR (N=1447).

Metabolite	HR (95% CI)	Raw *P* value
Cholesterol in very small VLDL	1.297 (1.057, 1.593)	0.0129
Cholesterol in small HDL	0.817 (0.687, 0.97)	0.0214
Triglycerides in IDL	1.222 (1.001, 1.491)	0.0492
Triglycerides in very large HDL	1.269 (1.035, 1.556)	0.0221
Triglycerides in large HDL	1.287 (1.053, 1.573)	0.0137
Phospholipids in very small VLDL	1.305 (1.059, 1.607)	0.0123
Cholesteryl esters in very small VLDL	1.311 (1.033, 1.665)	0.0259
Free cholesterol in very small VLDL	1.301 (1.058, 1.598)	0.0124
Total lipids in very small VLDL	1.301 (1.059, 1.599)	0.0122
Concentration of very small VLDL particles	1.269 (1.033, 1.557)	0.0231
Concentration of small HDL particles	0.791 (0.654, 0.957)	0.0158
Leucine	0.818 (0.67, 0.998)	0.0474
Albumin	0.601 (0.508, 0.712)	<0.0001

Estimated by Cox regression.

Adjusted for age, male sex, ever smoking, diabetes duration, systolic blood pressure, body mass index, glycated hemoglobin, oral antihyperglycemic drugs, insulin, antihypertensive drugs, lipid-lowering drugs, renin-angiotensin system blockers, statins, diabetic retinopathy, eGFR and ln(UACR).

Metabolites were $\log_e$-transformed and scaled to standard deviation.

ESM Table 11. Further adjustment for SGLT2i use during follow-up for metabolites associated with incident CVD (N=1447).

Metabolite	HR (95% CI)	Raw *P* value	FDR
Non-HDL-C	1.255 (1.035, 1.521)	0.0209	0.0253
Remnant cholesterol	1.31 (1.084, 1.583)	0.0052	0.0195
VLDL cholesterol	1.296 (1.071, 1.567)	0.0076	0.0222
Cholesterol in chylomicrons and extremely large VLDL	1.346 (1.053, 1.719)	0.0176	0.0244
Cholesterol in very large VLDL	1.365 (1.053, 1.77)	0.0189	0.0244
Cholesterol in large VLDL	1.261 (1.033, 1.539)	0.0224	0.026
Cholesterol in small VLDL	1.286 (1.058, 1.563)	0.0116	0.0229
Cholesterol in very small VLDL	1.459 (1.192, 1.785)	2.00E-04	0.0025
Cholesterol in medium HDL	0.839 (0.719, 0.98)	0.0266	0.027
Cholesterol in small HDL	0.774 (0.655, 0.914)	0.0025	0.0122
Total triglycerides	1.232 (1.028, 1.477)	0.0241	0.0262
Triglycerides in LDL	1.327 (1.114, 1.581)	0.0016	0.0109
Triglycerides in HDL	1.255 (1.05, 1.5)	0.0127	0.0238
Triglycerides in chylomicrons and extremely large VLDL	1.328 (1.049, 1.681)	0.0184	0.0244
Triglycerides in small VLDL	1.25 (1.041, 1.501)	0.0169	0.0244
Triglycerides in very small VLDL	1.332 (1.109, 1.601)	0.0022	0.0118
Triglycerides in IDL	1.372 (1.146, 1.642)	6.00E-04	0.0064
Triglycerides in large LDL	1.357 (1.134, 1.624)	9.00E-04	0.0075
Triglycerides in medium LDL	1.31 (1.099, 1.562)	0.0026	0.0122
Triglycerides in small LDL	1.259 (1.059, 1.497)	0.009	0.0227
Triglycerides in very large HDL	1.383 (1.117, 1.713)	0.003	0.0132
Triglycerides in large HDL	1.306 (1.05, 1.624)	0.0164	0.0244
Triglycerides in small HDL	1.231 (1.026, 1.478)	0.0254	0.0265
Phospholipids in VLDL	1.275 (1.06, 1.534)	0.0101	0.0227
Phospholipids in chylomicrons and extremely large VLDL	1.322 (1.049, 1.665)	0.018	0.0244
Phospholipids in very large VLDL	1.394 (1.081, 1.796)	0.0103	0.0227
Phospholipids in large VLDL	1.257 (1.031, 1.533)	0.0235	0.026
Phospholipids in small VLDL	1.269 (1.05, 1.532)	0.0136	0.0243
Phospholipids in very small VLDL	1.48 (1.224, 1.788)	1.00E-04	0.0019
Phospholipids in IDL	1.278 (1.042, 1.566)	0.0183	0.0244
Phospholipids in small LDL	1.342 (1.113, 1.618)	0.002	0.0118
Phospholipids in medium HDL	0.786 (0.654, 0.944)	0.01	0.0227

Cholesteryl esters in VLDL	1.311 (1.08, 1.591)	0.0062	0.0202
Cholesteryl esters in chylomicrons and extremely large VLDL	1.348 (1.052, 1.728)	0.0181	0.0244
Cholesteryl esters in large VLDL	1.265 (1.032, 1.55)	0.0234	0.026
Cholesteryl esters in small VLDL	1.308 (1.074, 1.594)	0.0077	0.0222
Cholesteryl esters in very small VLDL	1.478 (1.152, 1.896)	0.0021	0.0118
Cholesteryl esters in medium HDL	0.857 (0.745, 0.985)	0.0296	0.0296
Cholesteryl esters in small HDL	0.852 (0.771, 0.941)	0.0016	0.0109
Total free cholesterol	1.248 (1.032, 1.508)	0.022	0.026
Free cholesterol in VLDL	1.273 (1.055, 1.536)	0.0116	0.0229
Free cholesterol in chylomicrons and extremely large VLDL	1.327 (1.047, 1.683)	0.0194	0.0244
Free cholesterol in very large VLDL	1.381 (1.074, 1.777)	0.0119	0.0229
Free cholesterol in large VLDL	1.257 (1.031, 1.533)	0.0236	0.026
Free cholesterol in small VLDL	1.248 (1.029, 1.515)	0.0246	0.0262
Free cholesterol in very small VLDL	1.475 (1.219, 1.785)	1.00E-04	0.0019
Total lipids in VLDL	1.254 (1.043, 1.509)	0.0163	0.0244
Total lipids in chylomicrons and extremely large VLDL	1.332 (1.053, 1.686)	0.0169	0.0244
Total lipids in very large VLDL	1.299 (1.041, 1.621)	0.0204	0.0251
Total lipids in small VLDL	1.278 (1.06, 1.541)	0.0103	0.0227
Total lipids in very small VLDL	1.474 (1.218, 1.783)	1.00E-04	0.0019
Total lipids in IDL	1.288 (1.053, 1.576)	0.0136	0.0243
Total lipids in small LDL	1.26 (1.044, 1.519)	0.0157	0.0244
Total lipids in medium HDL	0.793 (0.662, 0.949)	0.0116	0.0229
Concentration of VLDL particles	1.296 (1.078, 1.558)	0.0058	0.0198
Concentration of HDL particles	0.764 (0.622, 0.937)	0.0098	0.0227
Concentration of chylomicrons and extremely large VLDL particles	1.323 (1.05, 1.667)	0.0174	0.0244
Concentration of very large VLDL particles	1.319 (1.05, 1.657)	0.0172	0.0244
Concentration of small VLDL particles	1.281 (1.063, 1.544)	0.0093	0.0227
Concentration of very small VLDL particles	1.438 (1.19, 1.738)	2.00E-04	0.0025
Concentration of IDL particles	1.272 (1.035, 1.565)	0.0225	0.026
Concentration of small LDL particles	1.265 (1.054, 1.518)	0.0117	0.0229
Concentration of medium HDL particles	0.791 (0.655, 0.954)	0.0144	0.0244
Concentration of small HDL particles	0.764 (0.635, 0.918)	0.0042	0.0166
Apolipoprotein B	1.258 (1.038, 1.525)	0.0195	0.0244
Ratio of apolipoprotein B to apolipoprotein A1	1.306 (1.081, 1.577)	0.0056	0.0198
Ratio of docosahexaenoic acid to total FAs	0.798 (0.662, 0.962)	0.0181	0.0244

Glycine	1.252 (1.029, 1.523)	0.0248	0.0262
Leucine	0.764 (0.629, 0.929)	0.0069	0.0216
Valine	0.802 (0.66, 0.975)	0.0265	0.027
Phenylalanine	1.272 (1.041, 1.555)	0.0187	0.0244
Tyrosine	0.778 (0.644, 0.94)	0.0094	0.0227
Creatinine	1.439 (1.167, 1.774)	7.00E-04	0.0066
Albumin	0.547 (0.474, 0.633)	0	0
Glycoprotein acetyls	1.288 (1.086, 1.528)	0.0036	0.015

Estimated by Cox regression.

Adjusted for age, male sex, ever smoking, diabetes duration, systolic blood pressure, body mass index, glycated hemoglobin, oral antihyperglycemic drugs, insulin, antihypertensive drugs, lipid-lowering drugs, renin-angiotensin system blockers, statins, diabetic retinopathy, CKD, severely increased albuminuria, and SGLT2i use during follow-up.

Metabolites were $\log_e$-transformed and scaled to standard deviation.

ESM Table 12. Associations of metabolites with CKD across different studies.

	HKDB		European cohorts[1]			Mexican cohort[2]	
**Metabolite**	**Beta (95% CI)**	**FDR**	**Beta**	**Se**	**FDR**	**Beta (95% CI)**	**FDR**
Total cholesterol	0.217 (0.096, 0.338)	0.000652459	0.125761833	0.087818757	0.182072437	NA	NA
Non-HDL-C	0.3439 (0.2222, 0.4655)	9.62E-08	NA	NA	NA	NA	NA
Remnant cholesterol	0.4034 (0.2827, 0.5242)	4.16E-10	0.389705377	0.091925613	0.000110542	NA	NA
VLDL cholesterol	0.429 (0.3089, 0.5492)	2.86E-11	0.353905518	0.073644401	1.97E-05	NA	NA
LDL cholesterol	0.264 (0.141, 0.3869)	4.55E-05	0.140148251	0.116532081	0.26788089	NA	NA
HDL cholesterol	-0.4452 (-0.5583, -0.3321)	3.23E-13	-0.380324659	0.086739428	7.06E-05	NA	NA
Cholesterol in chylomicrons and extremely large VLDL	0.1733 (0.0511, 0.2955)	0.006549292	0.242384968	0.075844011	0.003071428	-0.11 (-0.22, 0.01)	no
Cholesterol in very large VLDL	0.3243 (0.2026, 0.4459)	4.63E-07	0.25212045	0.074763095	0.00174343	-0.17 (-0.28, -0.05)	yes
Cholesterol in large VLDL	0.3626 (0.243, 0.4821)	1.26E-08	0.272308683	0.073855534	0.000604995	-0.09 (-0.20, 0.03)	no
Cholesterol in medium VLDL	0.3682 (0.2445, 0.4918)	2.11E-08	0.313564272	0.072839733	9.53E-05	0.13 (0.02, 0.25)	yes
Cholesterol in small VLDL	0.4892 (0.3686, 0.6097)	7.26E-14	0.393894933	0.074766404	3.49E-06	0.56 (0.45, 0.67)	yes
Cholesterol in very small VLDL	0.3599 (0.2393, 0.4805)	2.06E-08	0.342430096	0.084733394	0.000203185	0.49 (0.38, 0.60)	yes
Cholesterol in IDL	0.1877 (0.0674, 0.3079)	0.002935298	0.206659547	0.099665267	0.053653747	0.30 (0.19, 0.42)	yes
Cholesterol in large LDL	0.2305 (0.1076, 0.3533)	0.000364622	0.165442987	0.09870782	0.114883768	0.33 (0.22, 0.45)	yes
Cholesterol in medium LDL	0.2974 (0.1749, 0.42)	4.01E-06	0.206323055	0.083637988	0.021581518	0.36 (0.24, 0.47)	yes
Cholesterol in small LDL	0.3195 (0.1976, 0.4414)	6.97E-07	0.196512619	0.084002533	0.027999055	0.35 (0.23, 0.46)	yes
Cholesterol in very large HDL	-0.2288 (-0.3511, -0.1066)	0.000372377	-0.303501623	0.071049656	0.000101684	-0.01 (-0.12, 0.11)	no
Cholesterol in large HDL	-0.3522 (-0.4738, -0.2307)	4.77E-08	-0.349720296	0.078198516	5.35E-05	-0.18 (-0.29, -0.07)	yes
Cholesterol in medium HDL	-0.478 (-0.5934, -0.3627)	2.72E-14	-0.423548827	0.106398047	0.000242281	-0.55 (-0.67, -0.44)	yes
Cholesterol in small HDL	-0.2308 (-0.3514, -0.1101)	0.000279556	-0.096859613	0.11680265	0.465094904	0.22 (0.11, 0.33)	yes
Total triglycerides	0.2969 (0.177, 0.4168)	2.66E-06	0.278398045	0.072323934	0.000375019	NA	NA
Triglycerides in VLDL	0.2781 (0.1582, 0.3981)	1.07E-05	0.276102239	0.072776655	0.000442936	NA	NA
Triglycerides in LDL	0.3886 (0.2704, 0.5068)	7.48E-10	0.171278763	0.074925087	0.03191175	NA	NA
Triglycerides in HDL	0.3164 (0.1965, 0.4363)	6.06E-07	0.234615565	0.073871825	0.003242602	NA	NA
Triglycerides in chylomicrons and extremely large VLDL	0.1639 (0.0421, 0.2856)	0.009757557	0.203262365	0.075679956	0.012787994	-0.30 (-0.41, -0.18)	yes
Triglycerides in very large VLDL	0.2482 (0.1281, 0.3683)	8.72E-05	0.230897393	0.075000609	0.004330353	-0.26 (-0.37, -0.14)	yes
Triglycerides in large VLDL	0.2392 (0.1187, 0.3597)	0.000163151	0.255259202	0.073963914	0.001368591	-0.19 (-0.31, -0.08)	yes
Triglycerides in medium VLDL	0.2984 (0.1771, 0.4197)	3.01E-06	0.274548165	0.072896129	0.000475185	-0.07 (-0.18, 0.05)	no
Triglycerides in small VLDL	0.356 (0.2366, 0.4755)	2.06E-08	0.294555326	0.072213039	0.000190375	0.24 (0.13, 0.36)	yes
Triglycerides in very small VLDL	0.4364 (0.3196, 0.5531)	3.67E-12	0.336785111	0.071676277	2.74E-05	0.54 (0.43, 0.66)	yes
Triglycerides in IDL	0.4042 (0.287, 0.5214)	1.12E-10	0.301215566	0.074559803	0.000203185	0.58 (0.47, 0.69)	yes
Triglycerides in large LDL	0.378 (0.2596, 0.4964)	2.07E-09	0.183709328	0.075748603	0.02372725	0.61 (0.50, 0.73)	yes

Triglycerides in medium LDL	0.3828 (0.2639, 0.5017)	1.56E-09	0.130400606	0.077034202	0.11183825	0.60 (0.49, 0.71)	yes
Triglycerides in small LDL	0.3579 (0.2384, 0.4774)	1.89E-08	0.227101405	0.076485676	0.005893938	0.42 (0.30, 0.53)	yes
Triglycerides in very large HDL	0.1866 (0.0573, 0.3159)	0.005761212	0.073624057	0.118208633	0.587507837	0.01 (-0.11, 0.12)	no
Triglycerides in large HDL	-0.1452 (-0.2722, -0.0182)	0.02858897	0.027740207	0.144800635	0.853691383	0.04 (-0.07, 0.15)	no
Triglycerides in medium HDL	0.2026 (0.0792, 0.3259)	0.001767579	0.131614941	0.07551285	0.102182651	-0.06 (-0.17, 0.05)	no
Triglycerides in small HDL	0.3381 (0.2199, 0.4562)	6.63E-08	0.276776565	0.073056123	0.000442936	0.20 (0.08, 0.31)	yes
Total phospholipids in lipoprotein particles	0.0918 (-0.0283, 0.212)	0.146945267	NA	NA	NA	NA	NA
Phospholipids in VLDL	0.383 (0.2635, 0.5025)	1.84E-09	NA	NA	NA	NA	NA
Phospholipids in LDL	0.2379 (0.1155, 0.3602)	0.00022122	NA	NA	NA	NA	NA
Phospholipids in HDL	-0.3911 (-0.5052, -0.2771)	1.40E-10	NA	NA	NA	NA	NA
Phospholipids in chylomicrons and extremely large VLDL	0.1857 (0.0645, 0.3068)	0.003432787	0.18833752	0.075331719	0.020462785	-0.20 (-0.32, -0.09)	yes
Phospholipids in very large VLDL	0.3027 (0.1823, 0.4231)	1.92E-06	0.214738579	0.075105685	0.007971088	-0.21 (-0.32, -0.09)	yes
Phospholipids in large VLDL	0.3206 (0.2015, 0.4397)	3.60E-07	0.255783846	0.074221548	0.001371584	-0.13 (-0.25, -0.02)	yes
Phospholipids in medium VLDL	0.3715 (0.2494, 0.4935)	1.15E-08	0.289920865	0.072759422	0.000242281	0.04 (-0.08, 0.15)	no
Phospholipids in small VLDL	0.4482 (0.3281, 0.5684)	3.79E-12	0.308804637	0.071781559	9.53E-05	0.48 (0.37, 0.59)	yes
Phospholipids in very small VLDL	0.4845 (0.367, 0.6019)	3.09E-14	0.308017689	0.080123102	0.000375019	0.55 (0.44, 0.66)	yes
Phospholipids in IDL	0.1727 (0.0517, 0.2937)	0.006227789	0.161416209	0.085732701	0.078946902	0.41 (0.30, 0.52)	yes
Phospholipids in large LDL	0.1806 (0.058, 0.3031)	0.004903032	0.15720869	0.091562861	0.107133383	0.37 (0.26, 0.49)	yes
Phospholipids in medium LDL	0.2547 (0.1321, 0.3772)	8.00E-05	0.216316113	0.086753983	0.020462785	0.32 (0.21, 0.44)	yes
Phospholipids in small LDL	0.3392 (0.2183, 0.4602)	1.21E-07	0.157184508	0.0879956	0.093827158	0.26 (0.14, 0.37)	yes
Phospholipids in very large HDL	-0.2105 (-0.3356, -0.0853)	0.001369334	-0.35265142	0.074720322	2.74E-05	0.09 (-0.02, 0.21)	no
Phospholipids in large HDL	-0.3881 (-0.5052, -0.271)	5.58E-10	-0.369909855	0.090143641	0.000187393	-0.13 (-0.24, -0.02)	yes
Phospholipids in medium HDL	-0.4123 (-0.5291, -0.2956)	4.36E-11	-0.420092208	0.080398233	3.78E-06	-0.40 (-0.51, -0.29)	yes
Phospholipids in small HDL	-0.2057 (-0.326, -0.0854)	0.001136196	-0.290068527	0.069169824	0.000130434	-0.50 (-0.61, -0.38)	yes
Total esterified cholesterol	0.1838 (0.063, 0.3045)	0.003640348	0.067035248	0.094107904	0.536238692	NA	NA
Cholesteryl esters in VLDL	0.4575 (0.3369, 0.5781)	1.71E-12	NA	NA	NA	NA	NA
Cholesteryl esters in LDL	0.2974 (0.1748, 0.42)	4.04E-06	NA	NA	NA	NA	NA
Cholesteryl esters in HDL	-0.4509 (-0.5657, -0.3361)	3.32E-13	NA	NA	NA	NA	NA
Cholesteryl esters in chylomicrons and extremely large VLDL	0.1694 (0.0468, 0.2919)	0.008007525	0.28139131	0.076618094	0.000629096	-0.02 (-0.14, 0.09)	no
Cholesteryl esters in very large VLDL	0.3225 (0.1995, 0.4455)	6.96E-07	0.273380208	0.074694387	0.000649817	-0.14 (-0.25, -0.02)	yes
Cholesteryl esters in large VLDL	0.392 (0.2717, 0.5124)	1.03E-09	0.291116746	0.073613294	0.000258867	-0.02 (-0.13, 0.10)	no
Cholesteryl esters in medium VLDL	0.194 (0.0685, 0.3195)	0.003176604	0.328062881	0.073709099	5.51E-05	0.23 (0.11, 0.34)	yes
Cholesteryl esters in small VLDL	0.5099 (0.3899, 0.63)	6.71E-15	0.420285262	0.077323519	1.66E-06	0.47 (0.36, 0.58)	yes
Cholesteryl esters in very small VLDL	0.2577 (0.1328, 0.3826)	8.83E-05	0.307667173	0.082504179	0.000540887	0.41 (0.30, 0.52)	yes
Cholesteryl esters in IDL	0.2072 (0.0872, 0.3272)	0.001042627	0.232013441	0.098004211	0.026960307	0.26 (0.14, 0.37)	yes
Cholesteryl esters in large LDL	0.2662 (0.1433, 0.389)	3.85E-05	0.182277468	0.099092587	0.0848194	0.31 (0.20, 0.43)	yes
Cholesteryl esters in medium LDL	0.319 (0.1968, 0.4411)	7.55E-07	0.209033051	0.08285388	0.019440551	0.26 (0.15, 0.37)	yes

Cholesteryl esters in small LDL	0.3397 (0.218, 0.4613)	1.34E-07	0.194778628	0.083278457	0.027999055	0.30 (0.18, 0.41)	yes
Cholesteryl esters in very large HDL	-0.2341 (-0.3571, -0.111)	0.000301092	-0.28649932	0.07033502	0.000190375	0.01 (-0.11, 0.12)	no
Cholesteryl esters in large HDL	-0.3423 (-0.4644, -0.2201)	1.21E-07	-0.346382241	0.084913907	0.000190375	-0.19 (-0.30, -0.08)	yes
Cholesteryl esters in medium HDL	-0.4671 (-0.5849, -0.3494)	2.58E-13	-0.434078015	0.101608899	0.000101684	-0.60 (-0.72, -0.49)	yes
Cholesteryl esters in small HDL	-0.2817 (-0.4041, -0.1593)	1.24E-05	-0.037618546	0.127420764	0.804884555	0.19 (0.08, 0.31)	yes
Total free cholesterol	0.2813 (0.1599, 0.4026)	1.07E-05	0.233526101	0.082094187	0.008242397	NA	NA
Free cholesterol in VLDL	0.3909 (0.271, 0.5108)	1.02E-09	NA	NA	NA	NA	NA
Free cholesterol in LDL	0.1128 (-0.0114, 0.237)	0.083911864	NA	NA	NA	NA	NA
Free cholesterol in HDL	-0.324 (-0.4373, -0.2108)	6.63E-08	NA	NA	NA	NA	NA
Free cholesterol in chylomicrons and extremely large VLDL	0.1775 (0.0557, 0.2993)	0.005370663	0.199687074	0.075680732	0.014547005	-0.22 (-0.34, -0.11)	yes
Free cholesterol in very large VLDL	0.3068 (0.186, 0.4276)	1.51E-06	0.225732874	0.074910785	0.00523641	-0.20 (-0.32, -0.09)	yes
Free cholesterol in large VLDL	0.3302 (0.211, 0.4494)	1.67E-07	0.252816575	0.074264484	0.001575585	-0.16 (-0.28, -0.05)	yes
Free cholesterol in medium VLDL	0.3838 (0.2612, 0.5064)	4.22E-09	0.294079828	0.072918613	0.00020417	0.03 (-0.09, 0.14)	no
Free cholesterol in small VLDL	0.4453 (0.3239, 0.5666)	8.45E-12	0.334906347	0.072491748	3.24E-05	0.49 (0.38, 0.61)	yes
Free cholesterol in very small VLDL	0.4667 (0.3481, 0.5852)	3.23E-13	0.363847578	0.097803267	0.000548596	0.57 (0.46, 0.68)	yes
Free cholesterol in IDL	0.1301 (0.0083, 0.2519)	0.040848872	0.146749256	0.100038294	0.171777949	0.34 (0.23, 0.46)	yes
Free cholesterol in large LDL	0.094 (-0.0288, 0.2168)	0.146945267	0.107510681	0.095624543	0.302707122	0.34 (0.23, 0.46)	yes
Free cholesterol in medium LDL	0.1745 (0.0509, 0.2982)	0.006774349	0.160725055	0.086581845	0.083020982	0.35 (0.24, 0.46)	yes
Free cholesterol in small LDL	0.1658 (0.0412, 0.2904)	0.010544082	0.175877802	0.08649596	0.058590737	0.28 (0.17, 0.40)	yes
Free cholesterol in very large HDL	-0.1826 (-0.3081, -0.0571)	0.005398041	-0.340407399	0.07317716	3.13E-05	-0.08 (-0.19, 0.04)	no
Free cholesterol in large HDL	-0.3564 (-0.4772, -0.2356)	2.69E-08	-0.35615389	0.073909079	1.97E-05	-0.14 (-0.25, -0.03)	yes
Free cholesterol in medium HDL	-0.3803 (-0.4953, -0.2654)	5.92E-10	-0.379611949	0.126497733	0.005382974	-0.36 (-0.47, -0.25)	yes
Free cholesterol in small HDL	0.0143 (-0.1063, 0.135)	0.825609147	-0.222734977	0.06935551	0.002995661	-0.33 (-0.44, -0.21)	yes
Total lipids in lipoprotein particles	0.2471 (0.1256, 0.3686)	0.000111995	NA	NA	NA	NA	NA
Total lipids in VLDL	0.3465 (0.2266, 0.4664)	5.09E-08	NA	NA	NA	NA	NA
Total lipids in LDL	0.2772 (0.1548, 0.3996)	1.70E-05	NA	NA	NA	NA	NA
Total lipids in HDL	-0.4008 (-0.5138, -0.2877)	3.84E-11	NA	NA	NA	NA	NA
Total lipids in chylomicrons and extremely large VLDL	0.1769 (0.0553, 0.2985)	0.005398041	0.208430986	0.075608164	0.010564591	-0.23 (-0.34, -0.11)	yes
Total lipids in very large VLDL	0.2929 (0.1728, 0.413)	3.66E-06	0.232545025	0.07485403	0.004050958	-0.23 (-0.34, -0.12)	yes
Total lipids in large VLDL	0.2969 (0.1771, 0.4166)	2.64E-06	0.259353948	0.07387797	0.001132763	-0.16 (-0.27, -0.04)	yes
Total lipids in medium VLDL	0.349 (0.227, 0.4709)	6.63E-08	0.288783947	0.072634871	0.000242281	0.01 (-0.11, 0.12)	no
Total lipids in small VLDL	0.437 (0.3175, 0.5564)	9.33E-12	0.33184417	0.071733454	3.24E-05	0.43 (0.32, 0.55)	yes
Total lipids in very small VLDL	0.4614 (0.3431, 0.5798)	4.74E-13	0.385958342	0.077201633	9.71E-06	0.62 (0.51, 0.73)	yes
Total lipids in IDL	0.2241 (0.1039, 0.3443)	0.000392785	0.215374113	0.097729636	0.039122503	0.41 (0.30, 0.52)	yes
Total lipids in large LDL	0.2384 (0.1158, 0.361)	0.00022122	0.174305006	0.094074156	0.083020982	0.39 (0.28, 0.51)	yes
Total lipids in medium LDL	0.2976 (0.1752, 0.4199)	3.89E-06	0.212924143	0.084373898	0.019440551	0.40 (0.28, 0.51)	yes
Total lipids in small LDL	0.3349 (0.2135, 0.4563)	1.85E-07	0.207195003	0.085276442	0.023680497	0.36 (0.24, 0.47)	yes

Total lipids in very large HDL	-0.2072 (-0.3281, -0.0864)	0.001107339	-0.332172764	0.07317631	4.31E-05	0.12 (0.01, 0.23)	yes
Total lipids in large HDL	-0.3761 (-0.4925, -0.2597)	1.41E-09	-0.353337682	0.089603296	0.000264738	-0.10 (-0.22, 0.01)	no
Total lipids in medium HDL	-0.4419 (-0.5571, -0.3266)	1.04E-12	-0.421430303	0.089813846	2.74E-05	-0.46 (-0.57, -0.35)	yes
Total lipids in small HDL	-0.1641 (-0.2846, -0.0437)	0.008920623	-0.206861966	0.068289053	0.0050364	-0.15 (-0.26, -0.03)	yes
Total concentration of lipoprotein particles	-0.2671 (-0.3837, -0.1506)	1.33E-05	NA	NA	NA	NA	NA
Concentration of VLDL particles	0.4173 (0.2978, 0.5367)	7.14E-11	NA	NA	NA	NA	NA
Concentration of LDL particles	0.3336 (0.2113, 0.4559)	2.49E-07	NA	NA	NA	NA	NA
Concentration of HDL particles	-0.3401 (-0.4558, -0.2244)	2.98E-08	NA	NA	NA	NA	NA
Concentration of chylomicrons and extremely large VLDL particles	0.1905 (0.0694, 0.3116)	0.002734205	0.207335852	0.07562639	0.010934046	-0.24 (-0.35, -0.12)	yes
Concentration of very large VLDL particles	0.2957 (0.1757, 0.4156)	2.92E-06	0.232075661	0.074892702	0.00410236	-0.24 (-0.35, -0.12)	yes
Concentration of large VLDL particles	0.305 (0.1854, 0.4245)	1.36E-06	0.258848323	0.073892454	0.001146175	-0.16 (-0.28, -0.05)	yes
Concentration of medium VLDL particles	0.3705 (0.2486, 0.4925)	1.20E-08	0.28621483	0.072663842	0.000264738	-0.01 (-0.12, 0.11)	no
Concentration of small VLDL particles	0.4317 (0.3127, 0.5507)	1.35E-11	0.325139381	0.071645139	4.31E-05	0.40 (0.29, 0.52)	yes
Concentration of very small VLDL particles	0.4616 (0.343, 0.5802)	4.78E-13	0.385137535	0.076275002	8.42E-06	0.63 (0.52, 0.74)	yes
Concentration of IDL particles	0.2928 (0.1712, 0.4145)	4.76E-06	0.232781055	0.093360986	0.020462785	0.43 (0.32, 0.54)	yes
Concentration of large LDL particles	0.3072 (0.1841, 0.4302)	2.26E-06	0.182071909	0.09243991	0.067545241	0.41 (0.30, 0.52)	yes
Concentration of medium LDL particles	0.3215 (0.1992, 0.4439)	6.64E-07	0.215097974	0.084202425	0.018159728	0.41 (0.29, 0.52)	yes
Concentration of small LDL particles	0.3594 (0.2382, 0.4807)	2.39E-08	0.211202166	0.08511948	0.020947978	0.36 (0.25, 0.48)	yes
Concentration of very large HDL particles	-0.1357 (-0.2585, -0.0128)	0.034542763	-0.330252869	0.073308199	4.80E-05	0.13 (0.02, 0.25)	yes
Concentration of large HDL particles	-0.3487 (-0.4657, -0.2318)	2.06E-08	-0.351361227	0.092742594	0.000442936	-0.10 (-0.21, 0.01)	no
Concentration of medium HDL particles	-0.3923 (-0.5072, -0.2773)	1.72E-10	-0.415609989	0.086506435	1.97E-05	-0.45 (-0.56, -0.34)	yes
Concentration of small HDL particles	-0.187 (-0.3075, -0.0665)	0.00308149	-0.198196957	0.068417479	0.007161073	-0.16 (-0.27, -0.05)	yes
Average diameter for VLDL particles	0.1439 (0.0231, 0.2646)	0.022433413	0.179107508	0.074143562	0.024113698	-0.46 (-0.57, -0.35)	yes
Average diameter for LDL particles	-0.0269 (-0.1499, 0.096)	0.683583043	0.040916954	0.071916634	0.622642537	-0.13 (-0.24, -0.02)	yes
Average diameter for HDL particles	-0.2531 (-0.3678, -0.1384)	2.79E-05	-0.340823498	0.076641119	5.51E-05	0.04 (-0.07, 0.15)	no
Phosphoglycerides	0.211 (0.0887, 0.3333)	0.001042627	0.034414186	0.078301364	0.714659852	NA	NA
Ratio of triglycerides to phosphoglycerides	0.2942 (0.1763, 0.4122)	2.26E-06	NA	NA	NA	NA	NA
Total cholines	0.2106 (0.0883, 0.3329)	0.001057918	0.050857311	0.081017048	0.587507837	0.60 (0.49, 0.72)	yes
Phosphatidylcholines	0.1936 (0.0714, 0.3159)	0.002569545	0.019413135	0.08075513	0.826333207	0.51 (0.40, 0.62)	yes
Sphingomyelins	0.1768 (0.0537, 0.3)	0.005971131	0.140189599	0.094225247	0.166348886	0.19 (0.07, 0.30)	yes
Apolipoprotein B	0.3603 (0.2385, 0.4821)	2.50E-08	0.30971291	0.076182599	0.000191799	0.37 (0.25, 0.48)	yes
Apolipoprotein A1	-0.3962 (-0.5103, -0.2821)	8.54E-11	-0.305720311	0.082263393	0.000548596	0.03 (-0.09, 0.14)	no
Ratio of apolipoprotein B to apolipoprotein A1	0.5075 (0.3882, 0.6268)	6.71E-15	NA	NA	NA	0.42 (0.31, 0.54)	yes
Ratio of omega-3 FAs to total FAs	-0.036 (-0.1595, 0.0875)	0.584418074	NA	NA	NA	-0.01 (-0.12, 0.11)	no
Ratio of omega-6 FAs to total FAs	-0.2485 (-0.3718, -0.1251)	0.000129925	NA	NA	NA	0.23 (0.12, 0.35)	yes
Ratio of polyunsaturated FAs to total FAs	-0.2732 (-0.3963, -0.1501)	2.51E-05	NA	NA	NA	0.23 (0.11, 0.34)	yes
Ratio of monounsaturated FAs to total FAs	0.4193 (0.2986, 0.5399)	8.54E-11	NA	NA	NA	0.19 (0.07, 0.30)	yes
Ratio of saturated FAs to total FAs	-0.0721 (-0.1972, 0.053)	0.277970174	NA	NA	NA	-0.66 (-0.77, -0.54)	yes

Ratio of linoleic acid to total FAs	-0.0529 (-0.1772, 0.0714)	0.426801946	NA	NA	NA	0.33 (0.22, 0.45)	yes
Ratio of docosahexaenoic acid to total FAs	-0.3413 (-0.4654, -0.2172)	2.02E-07	NA	NA	NA	0.13 (0.01, 0.24)	yes
Ratio of polyunsaturated FAs to monounsaturated FAs	-0.3628 (-0.4844, -0.2412)	2.06E-08	NA	NA	NA	NA	NA
Ratio of omega-6 FAs to omega-3 FAs	-0.0384 (-0.1613, 0.0845)	0.563532452	NA	NA	NA	NA	NA
Alanine	-0.07 (-0.1944, 0.0544)	0.288803418	-0.09310522	0.071354326	0.227941763	-0.24 (-0.36, -0.13)	yes
Glutamine	0.0402 (-0.0807, 0.161)	0.540058565	0.276157887	0.141820753	0.070532033	0.41 (0.30, 0.53)	yes
Glycine	0.3214 (0.2016, 0.4413)	3.93E-07	NA	NA	NA	NA	NA
Histidine	-0.0156 (-0.1444, 0.1132)	0.825609147	0.234514121	0.097651658	0.024816029	-0.01 (-0.12, 0.11)	no
Total concentration of BCAAs	-0.1869 (-0.303, -0.0708)	0.002180047	NA	NA	NA	NA	NA
Isoleucine	-0.0019 (-0.1193, 0.1154)	0.9741295	0.317848548	0.09920571	0.003030244	-0.32 (-0.43, -0.20)	yes
Leucine	-0.1782 (-0.2924, -0.0641)	0.002928066	0.168100377	0.182720507	0.411758649	-0.71 (-0.82, -0.60)	yes
Valine	-0.2569 (-0.3743, -0.1394)	3.29E-05	-0.031787116	0.13022242	0.826333207	-0.96 (-1.07, -0.85)	yes
Phenylalanine	0.3801 (0.2587, 0.5016)	4.22E-09	0.51779777	0.065532129	4.19E-13	0.30 (0.18, 0.42)	yes
Tyrosine	-0.3092 (-0.4297, -0.1888)	1.17E-06	-0.173710728	0.073880836	0.027883861	-0.94 (-1.05, -0.83)	yes
Glucose	-0.1823 (-0.2907, -0.0738)	0.001370374	NA	NA	NA	-0.13 (-0.25, -0.02)	yes
Lactate	-0.2138 (-0.337, -0.0905)	0.000994067	-0.31689094	0.077624394	0.000190375	-0.50 (-0.61, -0.39)	yes
Pyruvate	-0.076 (-0.201, 0.0489)	0.253934453	NA	NA	NA	NA	NA
Citrate	0.2569 (0.137, 0.3767)	4.65E-05	0.206477136	0.073768794	0.009388259	0.39 (0.27, 0.50)	yes
Glycerol	0.1042 (-0.0152, 0.2236)	0.096918964	NA	NA	NA	NA	NA
3-Hydroxybutyrate	-0.004 (-0.1293, 0.1212)	0.955157252	-0.009524415	0.132010891	0.942483567	0.33 (0.21, 0.45)	yes
Acetate	-0.0369 (-0.1634, 0.0896)	0.584418074	0.048586443	0.071953184	0.558283098	0.10 (-0.01, 0.22)	no
Acetoacetate	-0.0737 (-0.1983, 0.051)	0.266876282	0.163376141	0.091468035	0.093827158	-0.85 (-0.96, -0.74)	yes
Acetone	-0.0589 (-0.1833, 0.0656)	0.375828528	NA	NA	NA	NA	NA
Creatinine	1.2497 (1.1791, 1.3203)	1.58E-201	3.157123968	0.575663785	1.66E-06	NA	NA
Albumin	-0.2067 (-0.3195, -0.094)	0.000491016	0.035330006	0.144405649	0.826333207	-0.76 (-0.87, -0.65)	yes
Glycoprotein acetyls	0.5042 (0.39, 0.6184)	8.82E-16	0.353831378	0.083488769	0.000110542	0.34 (0.23, 0.46)	yes

HKDB: the Hong Kong Diabetes Biobank;

[1]: The study combined 5 cohorts of Europeans (Tofte N, et al. J Clin Endocrinol Metab 2020);

[2]: The Mexico City Prospective Study (Aguilar-Ramirez D, et al. J Clin Endocrinol Metab 2021).

ESM Table 13. Associations of metabolites with albuminuria across different studies.

	HKBD			European cohorts[1]		
**Metabolite**	**Beta (95% CI**	**Raw *P* value**	**FDR**	**Beta**	**Se**	**Raw *P* value**
Total cholesterol	0.2134 (0.086, 0.3408)	0.001038382	0.00166533	0.096688222	0.074919419	0.196855292
Non-HDL-C	0.2824 (0.1543, 0.4106)	1.61E-05	3.66E-05	NA	NA	NA
Remnant cholesterol	0.3497 (0.2225, 0.4768)	7.82E-08	2.77E-07	0.141896622	0.070327769	0.043628022
VLDL cholesterol	0.3874 (0.2609, 0.514)	2.31E-09	1.12E-08	0.134569629	0.065722762	0.040605623
LDL cholesterol	0.19 (0.0605, 0.3195)	0.004044985	0.005927995	0.043547196	0.072171822	0.54625443
HDL cholesterol	-0.36 (-0.4791, -0.241)	3.60E-09	1.65E-08	-0.04104827	0.067868234	0.545296596
Cholesterol in chylomicrons and extremely large VLDL	0.217 (0.0883, 0.3457)	0.000959306	0.001553162	0.094434257	0.066138758	0.153343603
Cholesterol in very large VLDL	0.2474 (0.1194, 0.3755)	0.000156103	0.000299307	0.093719309	0.065326678	0.151393886
Cholesterol in large VLDL	0.349 (0.2231, 0.4749)	6.15E-08	2.27E-07	0.092009201	0.064377153	0.152940627
Cholesterol in medium VLDL	0.1434 (0.0132, 0.2737)	0.030874795	0.041328466	0.121082813	0.06379966	0.057714547
Cholesterol in small VLDL	0.346 (0.219, 0.4729)	1.01E-07	3.50E-07	0.120578613	0.067735338	0.0750525
Cholesterol in very small VLDL	0.2 (0.073, 0.327)	0.002042651	0.003073015	0.057065729	0.088866109	0.520773185
Cholesterol in IDL	0.0361 (-0.0905, 0.1628)	0.576114633	0.615215992	0.032131269	0.089840748	0.72060754
Cholesterol in large LDL	0.1315 (0.0021, 0.2608)	0.046362156	0.059410035	0.032860834	0.071645341	0.646478521
Cholesterol in medium LDL	0.2518 (0.1228, 0.3808)	0.000133679	0.000261212	0.056682737	0.071883856	0.430385458
Cholesterol in small LDL	0.2797 (0.1513, 0.408)	2.02E-05	4.41E-05	0.015562139	0.070750308	0.82590339
Cholesterol in very large HDL	-0.3457 (-0.4744, -0.217)	1.54E-07	5.25E-07	-0.057031981	0.097279095	0.557692936
Cholesterol in large HDL	-0.4586 (-0.5867, -0.3306)	2.96E-12	4.20E-11	-0.024871601	0.067924836	0.714243024
Cholesterol in medium HDL	-0.4123 (-0.5337, -0.2908)	3.70E-11	3.14E-10	-0.053389492	0.066488603	0.421982173
Cholesterol in small HDL	-0.0667 (-0.1937, 0.0603)	0.30313922	0.350569166	-0.027875423	0.063124855	0.65878457
Total triglycerides	0.405 (0.2788, 0.5313)	3.90E-10	2.46E-09	0.117612269	0.062724338	0.060783654
Triglycerides in VLDL	0.3727 (0.2464, 0.4991)	8.42E-09	3.67E-08	0.096945831	0.063142987	0.124700812
Triglycerides in LDL	0.4677 (0.3432, 0.5921)	2.58E-13	6.27E-12	0.183198002	0.066268574	0.005701399
Triglycerides in HDL	0.4786 (0.3524, 0.6049)	1.60E-13	4.52E-12	0.13680826	0.062213368	0.027876707
Triglycerides in chylomicrons and extremely large VLDL	0.2203 (0.0921, 0.3485)	0.000768397	0.001280662	0.079892382	0.065431075	0.222080009
Triglycerides in very large VLDL	0.2778 (0.1513, 0.4043)	1.74E-05	3.88E-05	0.07660895	0.064570989	0.235452579
Triglycerides in large VLDL	0.3393 (0.2125, 0.4662)	1.74E-07	5.80E-07	0.074346728	0.063849233	0.244257623
Triglycerides in medium VLDL	0.3213 (0.1937, 0.449)	8.71E-07	2.51E-06	0.084759323	0.063167911	0.179657671
Triglycerides in small VLDL	0.4395 (0.3137, 0.5652)	9.85E-12	1.05E-10	0.10055466	0.062961876	0.11024962
Triglycerides in very small VLDL	0.4852 (0.3623, 0.6082)	1.63E-14	6.93E-13	0.14349147	0.062852277	0.022430629
Triglycerides in IDL	0.4772 (0.3539, 0.6006)	5.19E-14	1.76E-12	0.137541413	0.063813427	0.031133056
Triglycerides in large LDL	0.4426 (0.3179, 0.5673)	4.67E-12	5.67E-11	0.151945228	0.06588555	0.021099483

Triglycerides in medium LDL	0.455 (0.3298, 0.5802)	1.45E-12	2.74E-11	0.16601336	0.067205705	0.013502726
Triglycerides in small LDL	0.4498 (0.324, 0.5756)	3.30E-12	4.32E-11	0.125250278	0.0647945	0.053231426
Triglycerides in very large HDL	0.0503 (-0.0859, 0.1865)	0.468802054	0.517508761	-0.009359953	0.105300652	0.929171008
Triglycerides in large HDL	-0.1354 (-0.2691, -0.0017)	0.047207485	0.059890094	0.064636409	0.068313296	0.344058764
Triglycerides in medium HDL	0.3297 (0.1998, 0.4596)	7.01E-07	2.05E-06	0.078116055	0.064757766	0.227709203
Triglycerides in small HDL	0.4409 (0.3165, 0.5653)	5.03E-12	5.70E-11	0.123377204	0.063632566	0.052513473
Total phospholipids in lipoprotein particles	0.2958 (0.1693, 0.4223)	4.82E-06	1.24E-05	NA	NA	NA
Phospholipids in VLDL	0.4123 (0.2864, 0.5382)	1.68E-10	1.19E-09	NA	NA	NA
Phospholipids in LDL	0.1874 (0.0585, 0.3162)	0.004383601	0.006369335	NA	NA	NA
Phospholipids in HDL	-0.1351 (-0.2552, -0.015)	0.027471988	0.037065381	NA	NA	NA
Phospholipids in chylomicrons and extremely large VLDL	0.2353 (0.1078, 0.3629)	0.000304324	0.000544579	0.082999	0.065358081	0.204116041
Phospholipids in very large VLDL	0.2654 (0.1387, 0.3922)	4.18E-05	9.01E-05	0.088847674	0.064876804	0.170848374
Phospholipids in large VLDL	0.3523 (0.2269, 0.4777)	4.07E-08	1.61E-07	0.081735263	0.064268717	0.203453539
Phospholipids in medium VLDL	0.2853 (0.1568, 0.4138)	1.40E-05	3.22E-05	0.106081921	0.063292405	0.093726276
Phospholipids in small VLDL	0.3939 (0.2674, 0.5205)	1.24E-09	6.38E-09	0.12164488	0.063656933	0.056011684
Phospholipids in very small VLDL	0.4476 (0.3239, 0.5713)	1.81E-12	3.08E-11	0.125714409	0.070184732	0.073262308
Phospholipids in IDL	0.0441 (-0.0833, 0.1715)	0.497138719	0.541753733	0.00800053	0.069935869	0.908922221
Phospholipids in large LDL	0.0967 (-0.0323, 0.2257)	0.1418018	0.169762719	0.039215144	0.071620713	0.584008388
Phospholipids in medium LDL	0.2237 (0.0946, 0.3527)	0.000690537	0.001173912	0.151818829	0.073912017	0.039971049
Phospholipids in small LDL	0.3351 (0.2078, 0.4625)	2.73E-07	8.29E-07	0.059795784	0.068036322	0.379466174
Phospholipids in very large HDL	-0.351 (-0.4828, -0.2193)	1.94E-07	6.35E-07	-0.065369135	0.067474729	0.332647815
Phospholipids in large HDL	-0.4021 (-0.5253, -0.2788)	2.00E-10	1.31E-09	-0.015478054	0.068588157	0.821460761
Phospholipids in medium HDL	-0.1634 (-0.2863, -0.0405)	0.009214443	0.012735409	-0.039041949	0.067811677	0.564790399
Phospholipids in small HDL	0.1138 (-0.0129, 0.2404)	0.078369777	0.096542478	-0.006933687	0.065546669	0.91575496
Total esterified cholesterol	0.1727 (0.0456, 0.2998)	0.007782499	0.010934089	0.066025249	0.075652592	0.382803325
Cholesteryl esters in VLDL	0.3648 (0.2379, 0.4918)	2.01E-08	8.12E-08	NA	NA	NA
Cholesteryl esters in LDL	0.2446 (0.1155, 0.3737)	0.000209164	0.000382343	NA	NA	NA
Cholesteryl esters in HDL	-0.4068 (-0.5277, -0.2859)	5.37E-11	4.35E-10	NA	NA	NA
Cholesteryl esters in chylomicrons and extremely large VLDL	0.2086 (0.0796, 0.3376)	0.001546867	0.002412545	0.093823155	0.06747956	0.164409362
Cholesteryl esters in very large VLDL	0.2014 (0.0719, 0.3309)	0.002320165	0.003459895	0.090811353	0.065359993	0.164710318
Cholesteryl esters in large VLDL	0.3345 (0.2077, 0.4612)	2.52E-07	7.92E-07	0.092011476	0.064406302	0.153116406
Cholesteryl esters in medium VLDL	-0.0966 (-0.2288, 0.0355)	0.151616998	0.180243983	0.12509909	0.064960314	0.054132205
Cholesteryl esters in small VLDL	0.3491 (0.2226, 0.4755)	6.97E-08	2.52E-07	0.087484529	0.070376011	0.213830415
Cholesteryl esters in very small VLDL	0.0223 (-0.1092, 0.1538)	0.739809326	0.757636057	0.041097054	0.113788212	0.717971282
Cholesteryl esters in IDL	0.0683 (-0.058, 0.1947)	0.289058698	0.336575196	0.047809532	0.104631605	0.647719896
Cholesteryl esters in large LDL	0.1905 (0.0612, 0.3199)	0.003909217	0.005778843	0.047890007	0.075459653	0.525660707
Cholesteryl esters in medium LDL	0.2866 (0.158, 0.4152)	1.31E-05	3.05E-05	0.039948604	0.071408703	0.575864155

Cholesteryl esters in small LDL	0.3128 (0.1847, 0.441)	1.80E-06	5.03E-06	0.001976167	0.070556592	0.977655569
Cholesteryl esters in very large HDL	-0.3829 (-0.5125, -0.2533)	7.99E-09	3.57E-08	-0.052087305	0.104407721	0.617861353
Cholesteryl esters in large HDL	-0.4678 (-0.5964, -0.3392)	1.39E-12	2.74E-11	-0.023015057	0.067876184	0.734553899
Cholesteryl esters in medium HDL	-0.4461 (-0.5702, -0.3221)	2.44E-12	3.78E-11	-0.058573705	0.066147396	0.375885505
Cholesteryl esters in small HDL	-0.1854 (-0.3143, -0.0565)	0.004834602	0.006965104	-0.038730433	0.061461097	0.528588758
Total free cholesterol	0.2891 (0.1613, 0.4168)	9.65E-06	2.34E-05	0.146468866	0.074337577	0.048801629
Free cholesterol in VLDL	0.3944 (0.2682, 0.5207)	1.09E-09	5.77E-09	NA	NA	NA
Free cholesterol in LDL	-0.0344 (-0.1652, 0.0964)	0.60610711	0.628282278	NA	NA	NA
Free cholesterol in HDL	-0.12 (-0.2392, -7e-04)	0.048605386	0.061206783	NA	NA	NA
Free cholesterol in chylomicrons and extremely large VLDL	0.2237 (0.0954, 0.3519)	0.000637737	0.001095104	0.08411358	0.065576368	0.199603761
Free cholesterol in very large VLDL	0.264 (0.1368, 0.3912)	4.91E-05	0.000102946	0.095815574	0.065368193	0.142707465
Free cholesterol in large VLDL	0.3499 (0.2244, 0.4754)	5.18E-08	2.00E-07	0.089372391	0.064490104	0.165798486
Free cholesterol in medium VLDL	0.242 (0.1129, 0.3711)	0.000242983	0.000439437	0.110045555	0.063425578	0.082734724
Free cholesterol in small VLDL	0.331 (0.2032, 0.4588)	4.15E-07	1.24E-06	0.136721044	0.06424032	0.033314319
Free cholesterol in very small VLDL	0.3963 (0.2714, 0.5211)	5.95E-10	3.37E-09	0.085123762	0.067425138	0.206771458
Free cholesterol in IDL	-0.0547 (-0.1829, 0.0735)	0.402902107	0.453598399	-0.000174852	0.068727492	0.997970081
Free cholesterol in large LDL	-0.0789 (-0.2082, 0.0504)	0.23148352	0.273279156	-0.002831295	0.069021922	0.967279768
Free cholesterol in medium LDL	0.0571 (-0.0732, 0.1873)	0.390203427	0.442230551	0.090807235	0.071819943	0.20609599
Free cholesterol in small LDL	0.0633 (-0.0679, 0.1945)	0.344070558	0.395216181	0.037587185	0.068117069	0.58108351
Free cholesterol in very large HDL	-0.2894 (-0.4215, -0.1572)	1.85E-05	4.08E-05	-0.06741746	0.076163442	0.376065897
Free cholesterol in large HDL	-0.4336 (-0.5608, -0.3064)	3.01E-11	2.70E-10	-0.031144681	0.06799091	0.646901252
Free cholesterol in medium HDL	-0.1929 (-0.3139, -0.0718)	0.001809981	0.002747293	-0.032253058	0.067565881	0.633107809
Free cholesterol in small HDL	0.2639 (0.1368, 0.3909)	4.82E-05	0.000102452	0.0105531	0.066769848	0.874415919
Total lipids in lipoprotein particles	0.3757 (0.2478, 0.5037)	9.87E-09	4.19E-08	NA	NA	NA
Total lipids in VLDL	0.3958 (0.2696, 0.5221)	9.57E-10	5.25E-09	NA	NA	NA
Total lipids in LDL	0.2191 (0.0902, 0.348)	0.000875493	0.001431095	NA	NA	NA
Total lipids in HDL	-0.1906 (-0.3097, -0.0716)	0.001713064	0.002623612	NA	NA	NA
Total lipids in chylomicrons and extremely large VLDL	0.231 (0.1029, 0.359)	0.000412374	0.000722718	0.083283483	0.065486119	0.203453756
Total lipids in very large VLDL	0.284 (0.1576, 0.4104)	1.12E-05	2.67E-05	0.08274665	0.064659378	0.200639547
Total lipids in large VLDL	0.3506 (0.2245, 0.4768)	5.66E-08	2.14E-07	0.080133278	0.063974276	0.210356439
Total lipids in medium VLDL	0.2993 (0.1709, 0.4277)	5.16E-06	1.31E-05	0.099170963	0.063181148	0.116501447
Total lipids in small VLDL	0.4209 (0.2952, 0.5467)	6.80E-11	5.25E-10	0.118214267	0.063639567	0.063231628
Total lipids in very small VLDL	0.3977 (0.273, 0.5223)	4.87E-10	2.86E-09	0.132142252	0.068693186	0.054397365
Total lipids in IDL	0.099 (-0.0276, 0.2256)	0.125211865	0.150964661	0.082812608	0.072711934	0.25473921
Total lipids in large LDL	0.1529 (0.0237, 0.282)	0.020348051	0.027673349	0.050985494	0.071759009	0.477387925
Total lipids in medium LDL	0.262 (0.1332, 0.3908)	6.91E-05	0.0001382	0.098408952	0.072763604	0.176232211
Total lipids in small LDL	0.3173 (0.1894, 0.4451)	1.22E-06	3.46E-06	0.050899068	0.070866715	0.472611415

Total lipids in very large HDL	-0.3025 (-0.4298, -0.1752)	3.36E-06	8.93E-06	-0.07724625	0.066015103	0.241948677
Total lipids in large HDL	-0.4006 (-0.5233, -0.278)	1.86E-10	1.26E-09	-0.017376824	0.068506445	0.799764108
Total lipids in medium HDL	-0.2484 (-0.3697, -0.127)	6.21E-05	0.000127105	-0.038599379	0.067672985	0.568419372
Total lipids in small HDL	0.1102 (-0.0167, 0.2371)	0.088617571	0.108381202	0.006606167	0.068646442	0.9233342
Total concentration of lipoprotein particles	-0.058 (-0.1807, 0.0647)	0.353936806	0.403820517	NA	NA	NA
Concentration of VLDL particles	0.4359 (0.3101, 0.5617)	1.46E-11	1.38E-10	NA	NA	NA
Concentration of LDL particles	0.2486 (0.1199, 0.3774)	0.000156696	0.000299307	NA	NA	NA
Concentration of HDL particles	-0.1166 (-0.2384, 0.0052)	0.06058795	0.075734937	NA	NA	NA
Concentration of chylomicrons and extremely large VLDL particles	0.2427 (0.1152, 0.3701)	0.000194915	0.00036017	0.082499827	0.065469983	0.207627217
Concentration of very large VLDL particles	0.2908 (0.1645, 0.4171)	6.73E-06	1.68E-05	0.081197785	0.064631189	0.208998119
Concentration of large VLDL particles	0.3648 (0.2389, 0.4906)	1.52E-08	6.30E-08	0.078612547	0.063946483	0.218940971
Concentration of medium VLDL particles	0.2942 (0.1658, 0.4227)	7.44E-06	1.83E-05	0.096309608	0.063162151	0.127309147
Concentration of small VLDL particles	0.4342 (0.3089, 0.5596)	1.44E-11	1.38E-10	0.115164153	0.063393789	0.069271141
Concentration of very small VLDL particles	0.3987 (0.2738, 0.5236)	4.74E-10	2.86E-09	0.143406242	0.068045973	0.035075083
Concentration of IDL particles	0.1301 (0.002, 0.2582)	0.046479616	0.059410035	0.098566781	0.072753871	0.175482001
Concentration of large LDL particles	0.1803 (0.0507, 0.3099)	0.006428822	0.009184031	0.060579371	0.071834582	0.39905081
Concentration of medium LDL particles	0.2658 (0.137, 0.3946)	5.39E-05	0.000111774	0.105254222	0.072845864	0.148488932
Concentration of small LDL particles	0.3362 (0.2086, 0.4639)	2.66E-07	8.22E-07	0.057983194	0.070923201	0.413614744
Concentration of very large HDL particles	-0.2495 (-0.3789, -0.1201)	0.000160683	0.000300207	-0.076097226	0.066148688	0.249980559
Concentration of large HDL particles	-0.3808 (-0.504, -0.2577)	1.60E-09	7.98E-09	-0.015360677	0.068602384	0.822828275
Concentration of medium HDL particles	-0.2179 (-0.339, -0.0968)	0.000426157	0.000739252	-0.035165848	0.067725531	0.60359294
Concentration of small HDL particles	0.0018 (-0.1251, 0.1287)	0.977851102	0.977851102	0.013977585	0.068647451	0.838655123
Average diameter for VLDL particles	0.2451 (0.118, 0.3723)	0.000160699	0.000300207	0.042653944	0.064087798	0.505695686
Average diameter for LDL particles	-0.2625 (-0.392, -0.133)	7.28E-05	0.000143892	-0.025248071	0.060268793	0.675271345
Average diameter for HDL particles	-0.2011 (-0.3219, -0.0803)	0.001112595	0.001767674	-0.028874385	0.068335686	0.672632789
Phosphoglycerides	0.3106 (0.1815, 0.4396)	2.54E-06	6.96E-06	0.152677814	0.069228593	0.02742495
Ratio of triglycerides to phosphoglycerides	0.3778 (0.2534, 0.5022)	3.10E-09	1.46E-08	NA	NA	NA
Total cholines	0.3025 (0.1736, 0.4313)	4.43E-06	1.16E-05	0.15351577	0.072637248	0.034561588
Phosphatidylcholines	0.3409 (0.2121, 0.4697)	2.32E-07	7.45E-07	0.176422707	0.070519949	0.012358401
Sphingomyelins	0.2245 (0.0947, 0.3543)	0.00070586	0.00118808	0.078074148	0.069958658	0.264420386
Apolipoprotein B	0.287 (0.1588, 0.4152)	1.20E-05	2.83E-05	0.157567638	0.067414155	0.019423179
Apolipoprotein A1	-0.1269 (-0.247, -0.0068)	0.038417861	0.050628188	0.076709862	0.071078592	0.28048701
Ratio of apolipoprotein B to apolipoprotein A1	0.2992 (0.1736, 0.4248)	3.22E-06	8.69E-06	NA	NA	NA
Ratio of omega-3 FAs to total FAs	-0.0063 (-0.1369, 0.1243)	0.925134483	0.930608652	NA	NA	NA
Ratio of omega-6 FAs to total FAs	-0.1328 (-0.2633, -0.0023)	0.046052318	0.059410035	NA	NA	NA
Ratio of polyunsaturated FAs to total FAs	-0.1386 (-0.2688, -0.0085)	0.03687627	0.048976296	NA	NA	NA
Ratio of monounsaturated FAs to total FAs	0.1736 (0.046, 0.3013)	0.007705111	0.010915574	NA	NA	NA
Ratio of saturated FAs to total FAs	-0.0216 (-0.1539, 0.1107)	0.748979489	0.762434211	NA	NA	NA

Ratio of linoleic acid to total FAs	0.122 (-0.0094, 0.2535)	0.068846593	0.085430079	NA	NA	NA
Ratio of docosahexaenoic acid to total FAs	-0.2677 (-0.399, -0.1365)	6.60E-05	0.000133499	NA	NA	NA
Ratio of polyunsaturated FAs to monounsaturated FAs	-0.1626 (-0.2913, -0.034)	0.013231821	0.0181404	NA	NA	NA
Ratio of omega-6 FAs to omega-3 FAs	-0.0442 (-0.1737, 0.0853)	0.503554581	0.545250184	NA	NA	NA
Alanine	-0.0345 (-0.1655, 0.0966)	0.60610761	0.628282278	-0.00953934	0.149821376	0.94923186
Glutamine	-0.0447 (-0.1719, 0.0825)	0.491023934	0.53854238	-0.098682105	0.063380105	0.119473177
Glycine	0.0282 (-0.0981, 0.1546)	0.661142916	0.681177549	NA	NA	NA
Histidine	-0.045 (-0.1807, 0.0907)	0.515110611	0.554232936	-0.078857245	0.063449835	0.213931139
Total concentration of BCAAs	0.0136 (-0.1086, 0.1358)	0.827285049	0.83713368	NA	NA	NA
Isoleucine	0.2118 (0.0883, 0.3354)	0.000787849	0.001300334	0.020423058	0.06758375	0.762508077
Leucine	-0.0453 (-0.1655, 0.0749)	0.459739153	0.510821282	-0.023548793	0.068642064	0.731548428
Valine	-0.035 (-0.1587, 0.0887)	0.579026816	0.615215992	-0.008756442	0.06760516	0.896943601
Phenylalanine	0.1704 (0.0424, 0.2983)	0.009087947	0.012663533	0.017115668	0.099095066	0.862871773
Tyrosine	-0.4179 (-0.5448, -0.291)	1.34E-10	9.90E-10	-0.150587814	0.069838322	0.031065344
Glucose	0.1891 (0.0748, 0.3033)	0.001190674	0.001874208	0.304791557	0.09828353	0.001927733
Lactate	0.0343 (-0.0955, 0.1641)	0.604284965	0.628282278	0.106090858	0.161466813	0.511152398
Pyruvate	0.0508 (-0.0808, 0.1823)	0.449173484	0.502365081	NA	NA	NA
Citrate	0.2281 (0.1018, 0.3543)	0.000404008	0.000715431	-0.036962749	0.062126943	0.551873403
Glycerol	-0.0352 (-0.1611, 0.0908)	0.583827104	0.616463402	NA	NA	NA
3-Hydroxybutyrate	0.2118 (0.08, 0.3436)	0.00164825	0.002547295	0.026911594	0.061813392	0.663295081
Acetate	0.0754 (-0.0578, 0.2085)	0.267341788	0.3134352	-0.087788807	0.121512211	0.470006171
Acetoacetate	0.137 (0.0058, 0.2682)	0.04073932	0.053274495	0.064272094	0.07358847	0.382445652
Acetone	0.112 (-0.0191, 0.243)	0.094024522	0.114172634	NA	NA	NA
Creatinine	0.4551 (0.3806, 0.5295)	6.27E-32	1.07E-29	0.115795811	0.094054327	0.218263483
Albumin	-0.6215 (-0.7403, -0.5028)	4.33E-24	3.68E-22	0.054363803	0.061602498	0.377510006
Glycoprotein acetyls	0.5113 (0.391, 0.6316)	1.47E-16	8.32E-15	0.157293023	0.065908737	0.017008328

HKDB: the Hong Kong Diabetes Biobank;

[1]: The study combined 5 cohorts of Europeans (Tofte N, et al. J Clin Endocrinol Metab 2020).

ESM Fig. 1. Flow chart of study participants.

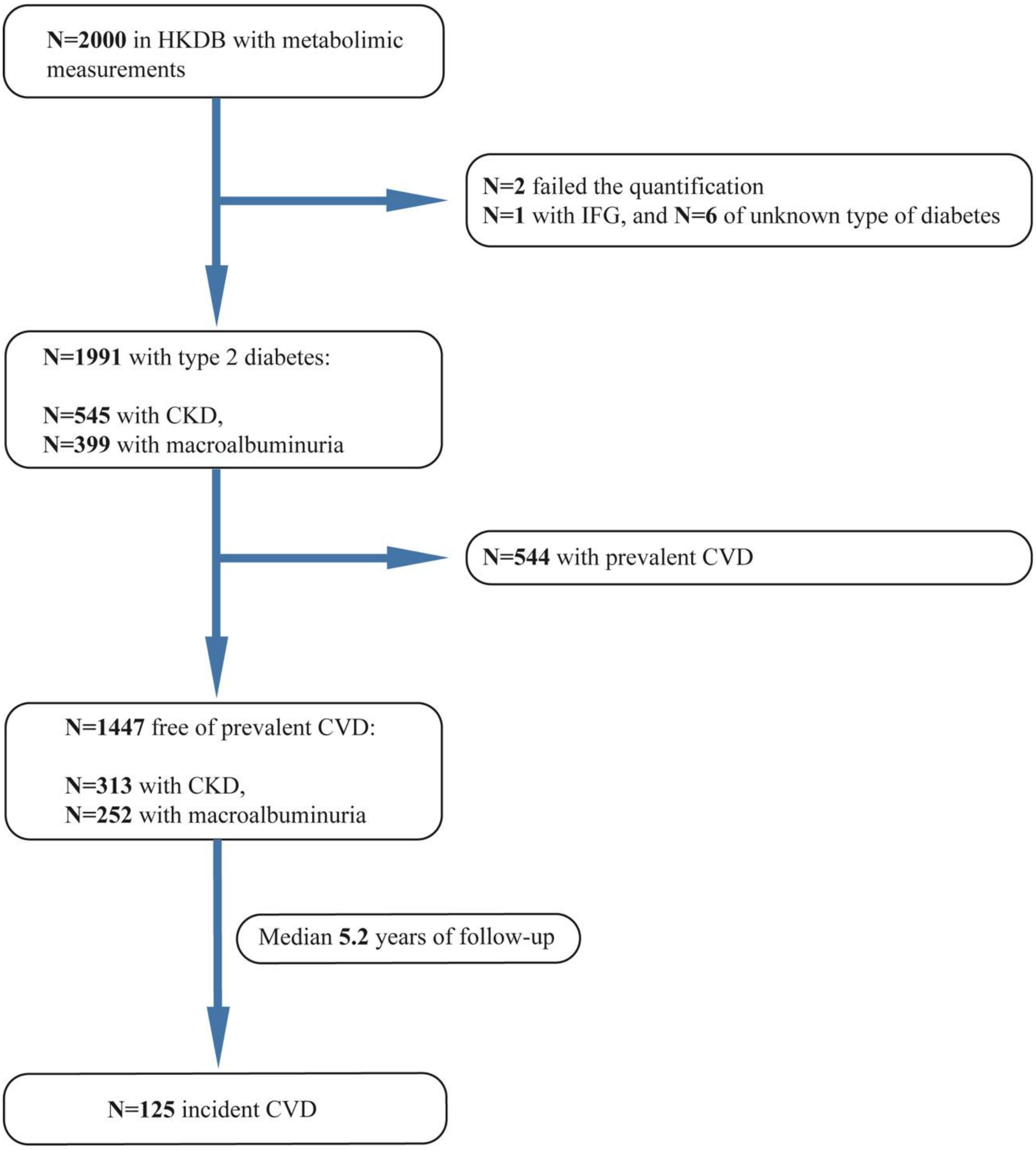

CKD, chronic kidney disease; CVD, cardiovascular disease; IFG, impaired fasting glucose; HKDB, the Hong Kong Diabetes Biobank

ESM Fig. 2. Correlation coefficients of metabolites measured by NMR and clinical biochemical assay.

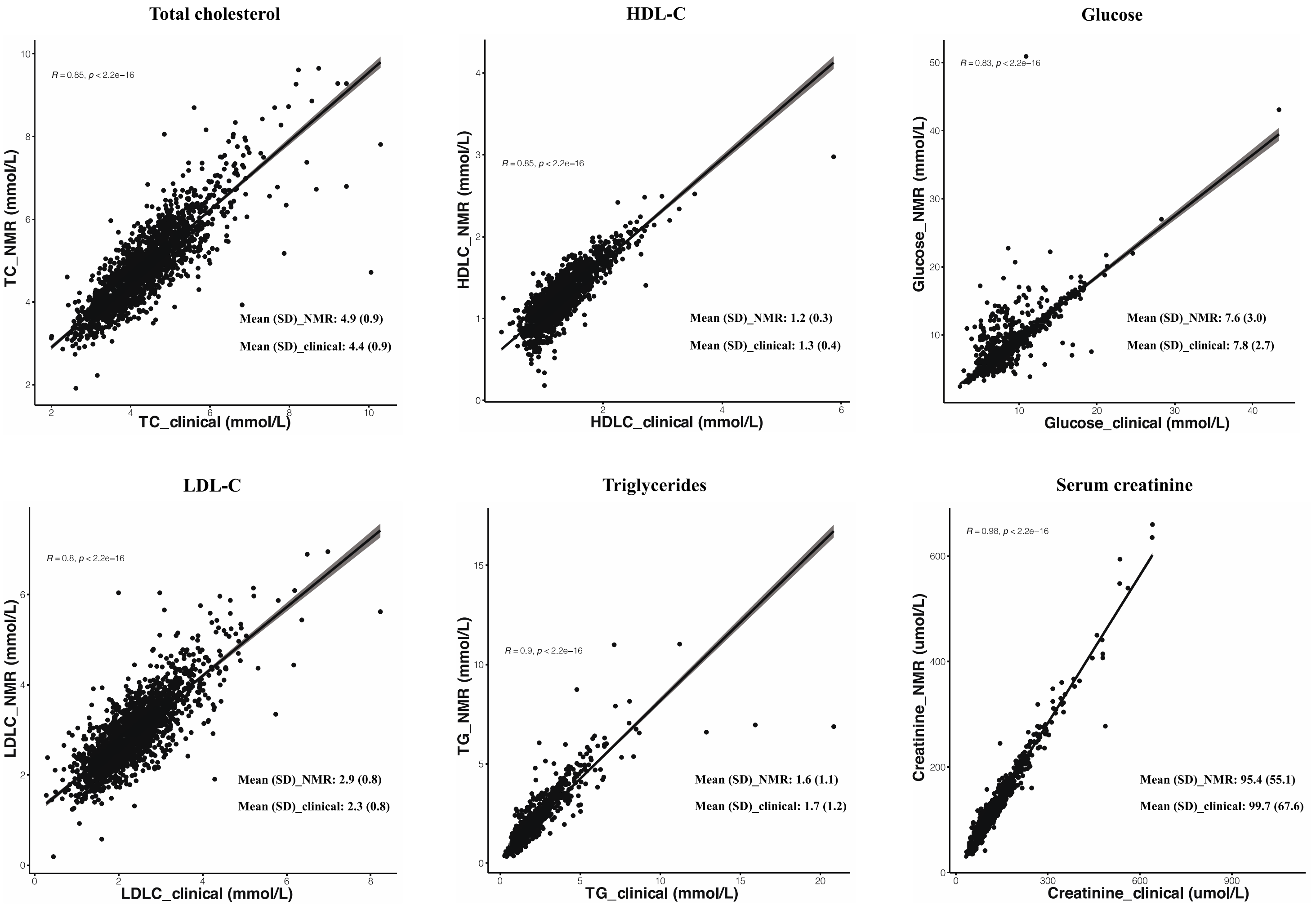

Estimated by Pearson correlation.

HDL-C, high-density lipoprotein cholesterol; LDL-C, low-density lipoprotein cholesterol; NMR, nuclear magnetic resonance; SD, standard deviation; TC, total cholesterol; TG, triglycerides.

ESM Fig. 3. Metabolites remaining associated with incident CVD after further adjustment for kidney function.

Measurements	HR (95% CI)	*P* value
**Lipoproteins**		
Cholesterol in chylomicrons and extremely large VLDL	1.293 (1.002, 1.668)	0.048
Cholesterol in very small VLDL	1.325 (1.084, 1.620)	0.006
Cholesterol in small HDL	0.815 (0.685, 0.971)	0.022
Triglycerides in LDL	1.226 (1.016, 1.480)	0.034
Triglycerides in chylomicrons and extremely large VLDL	1.283 (1.003, 1.641)	0.047
Triglycerides in IDL	1.263 (1.042, 1.530)	0.017
Triglycerides in large LDL	1.253 (1.035, 1.517)	0.021
Triglycerides in medium LDL	1.209 (1.000, 1.461)	0.0496
Triglycerides in very large HDL	1.301 (1.058, 1.599)	0.013
Triglycerides in large HDL	1.305 (1.063, 1.602)	0.011
Phospholipids in very small VLDL	1.337 (1.094, 1.634)	0.005
Phospholipids in small LDL	1.227 (1.014, 1.485)	0.035
Cholesteryl esters in chylomicrons and extremely large VLDL	1.299 (1.004, 1.681)	0.047
Cholesteryl esters in very small VLDL	1.341 (1.058, 1.698)	0.015
Cholesteryl esters in small HDL	0.890 (0.799, 0.990)	0.033
Free cholesterol in very small VLDL	1.332 (1.092, 1.625)	0.005
Total lipids in chylomicrons and extremely large VLDL	1.279 (1.001, 1.635)	0.049
Total lipids in very small VLDL	1.333 (1.092, 1.627)	0.005
Concentration of very small VLDL particles	1.303 (1.069, 1.588)	0.009
Concentration of small HDL particles	0.796 (0.657, 0.964)	0.020
**Amino acids**		
Leucine	0.810 (0.663, 0.990)	0.040
**Fluid balance**		
Albumin	0.583 (0.495, 0.687)	<0.0001

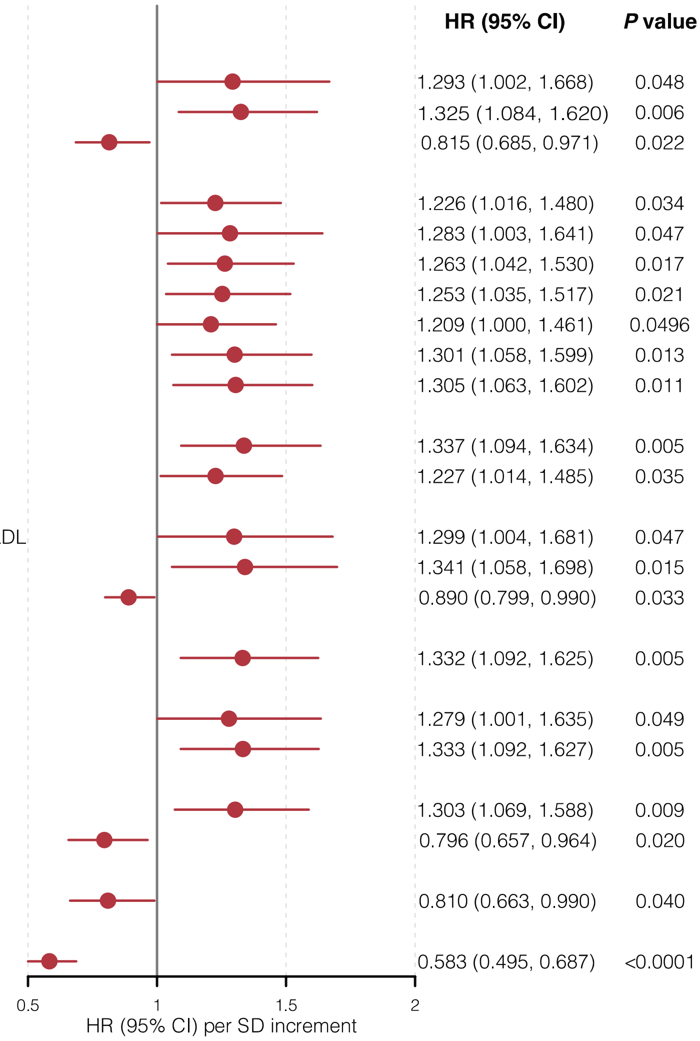

Estimated by Cox regression.
Adjusted for age, male sex, ever smoking, diabetes duration, systolic blood pressure, body mass index, glycated hemoglobin, oral antihyperglycemic drugs, insulin, antihypertensive drugs, lipid-lowering drugs, renin-angiotensin system blockers, statins, diabetic retinopathy, chronic kidney disease, and severely increased albuminuria.
Metabolites were log$_e$-transformed and scaled to standard deviation.

ESM Fig. 4. Receiver operating characteristic curves of metabolomic biomarkers for incident CVD in HKDB.

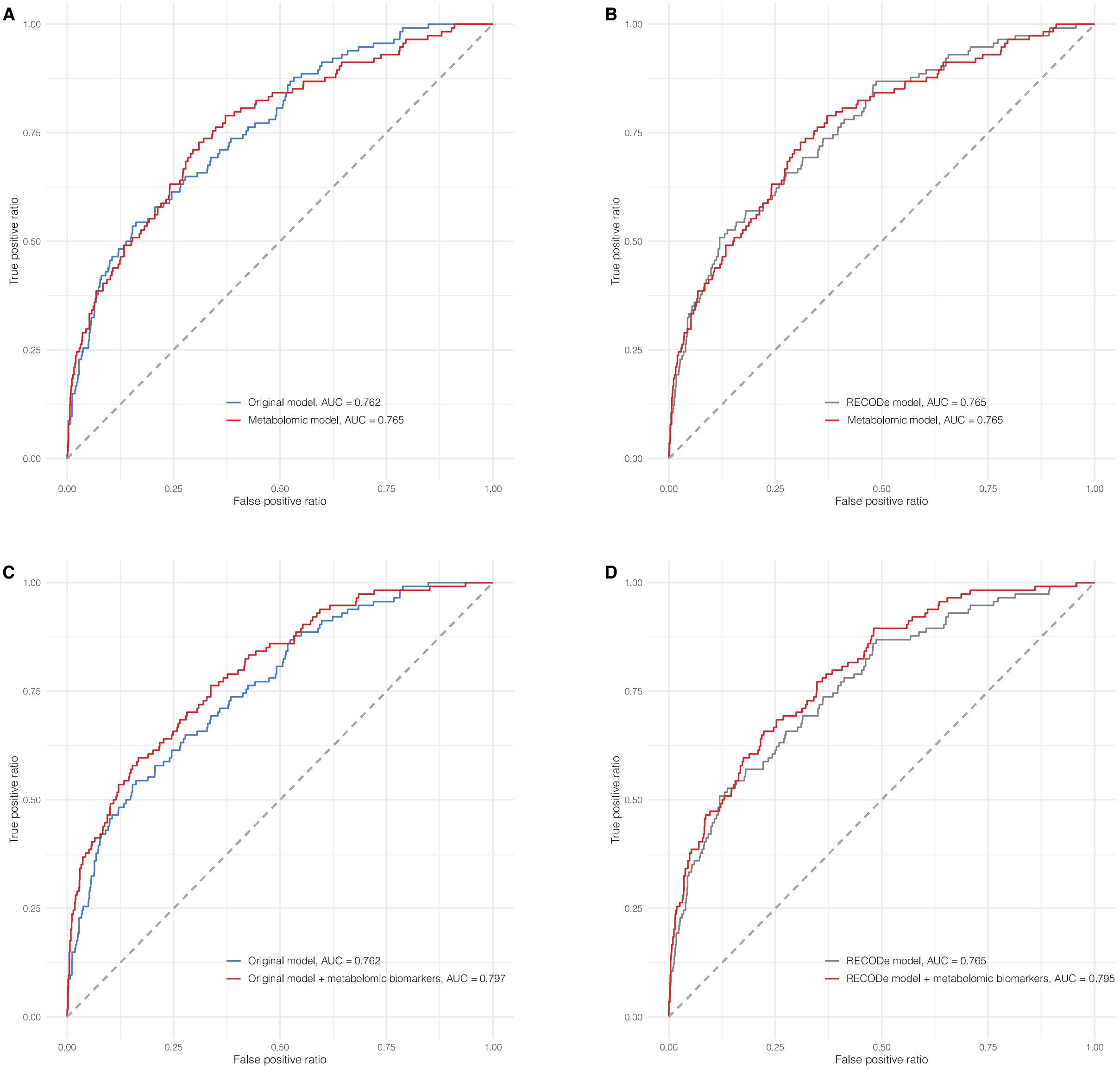

Receiver operating characteristic curves for metabolomic model and original model (**A**), metabolomic model and RECODe model (**B**), original model and adding metabolomic biomarkers (**C**), RECODe model and adding metabolomic biomarkers (**D**).

Metabolomic biomarkers: triglycerides in large HDL, phospholipids in small LDL, and albumin.

Metabolomic model: age, sex, triglycerides in large HDL, phospholipids in small LDL, and albumin.

Original model: age, male sex, ever smoking, diabetes duration, systolic blood pressure, body mass index, glycated hemoglobin, oral antihyperglycemic drugs, insulin, antihypertensive drugs, lipid-lowering drugs, renin-angiotensin system blockers, statins, diabetic retinopathy, severely increased albuminuria, and chronic kidney disease.

RECODe model: age, sex, ever smoking, systolic blood pressure, glycated hemoglobin, total cholesterol, high-density lipoprotein cholesterol, estimated glomerular filtration rate, ln (urinary albumin-creatinine ratio), antihypertensive drugs, and lipid-lowering drugs.
Metabolites were $\log_e$-transformed and scaled to standard deviation.
AUC, area under the receiver operating characteristic curve.

ESM Fig. 5. Receiver operating characteristic curves of metabolomic biomarkers for incident CVD in HKDR.

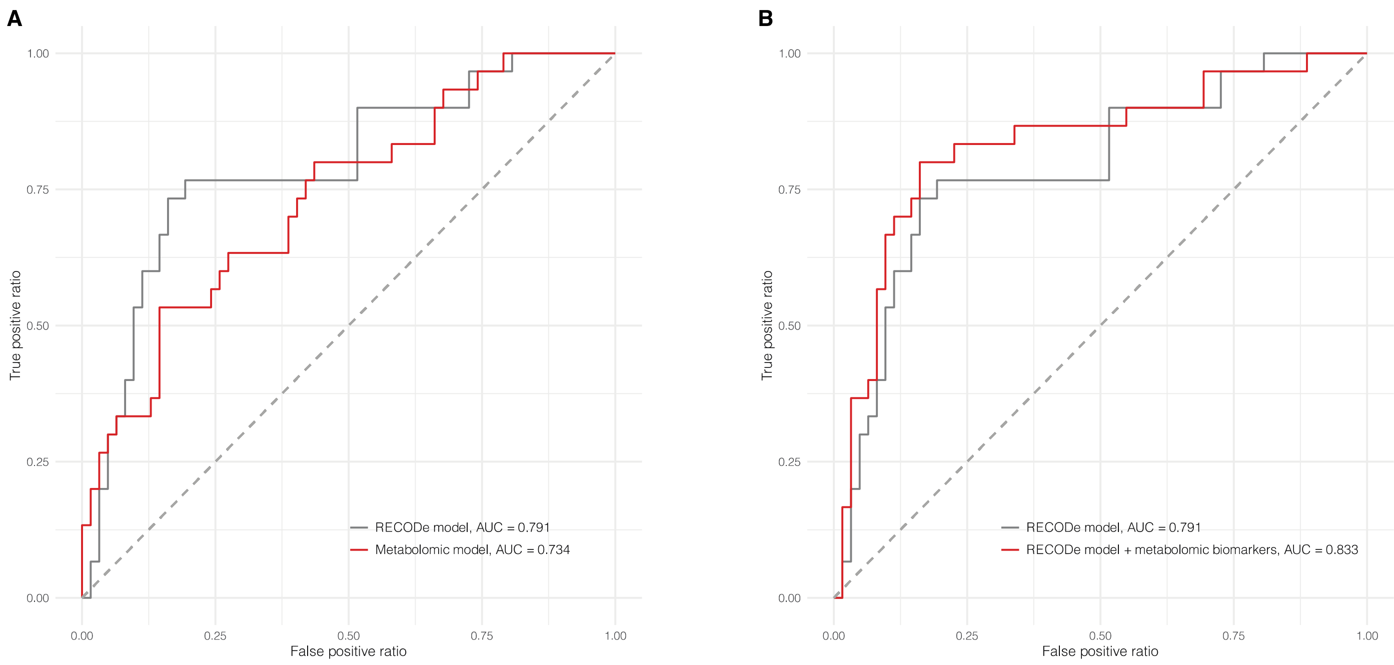

Receiver operating characteristic curves for metabolomic model and RECODe model (**A**), RECODe model and adding metabolomic biomarkers (**B**).
Metabolomic biomarkers: triglycerides in large HDL, phospholipids in small LDL, and albumin.
Metabolomic model: age, sex, triglycerides in large HDL, phospholipids in small LDL, and albumin.
RECODe model: age, sex, ever smoking, systolic blood pressure, glycated hemoglobin, total cholesterol, high-density lipoprotein cholesterol, estimated glomerular filtration rate, ln (urinary albumin-creatinine ratio), antihypertensive drugs, and lipid-lowering drugs.
Metabolites were $\log_e$-transformed and scaled to standard deviation.
AUC: area under the receiver operating characteristic curve.
